# Glucocorticoids unmask silent non-coding genetic risk variants for common diseases

**DOI:** 10.1093/nar/gkac1045

**Published:** 2022-11-18

**Authors:** Thanh Thanh L Nguyen, Huanyao Gao, Duan Liu, Trudy Janice Philips, Zhenqing Ye, Jeong-Heon Lee, Geng-xian Shi, Kaleigh Copenhaver, Lingxin Zhang, Lixuan Wei, Jia Yu, Huan Zhang, Abhijeet Barath, Maggie Luong, Cheng Zhang, Alexandre Gaspar-Maia, Hu Li, Liewei Wang, Tamas Ordog, Richard M Weinshilboum

**Affiliations:** Department of Molecular Pharmacology and Experimental Therapeutics, Mayo Clinic; Rochester, MN, USA; Mayo Clinic Graduate School of Biomedical Sciences, Mayo Clinic; Rochester, MN, USA; Department of Molecular Pharmacology and Experimental Therapeutics, Mayo Clinic; Rochester, MN, USA; Department of Molecular Pharmacology and Experimental Therapeutics, Mayo Clinic; Rochester, MN, USA; Department of Molecular Pharmacology and Experimental Therapeutics, Mayo Clinic; Rochester, MN, USA; Department of Health Sciences Research, Mayo Clinic; Rochester, MN, USA; Current affiliation: Greehey Children's Cancer Research Institute, University of Texas Health San Antonio; San Antonio, TX 78229, USA; Epigenomics Program, Center for Individualized Medicine, Mayo Clinic; Rochester, MN, USA; Epigenomics Program, Center for Individualized Medicine, Mayo Clinic; Rochester, MN, USA; Department of Molecular Pharmacology and Experimental Therapeutics, Mayo Clinic; Rochester, MN, USA; Department of Molecular Pharmacology and Experimental Therapeutics, Mayo Clinic; Rochester, MN, USA; Department of Molecular Pharmacology and Experimental Therapeutics, Mayo Clinic; Rochester, MN, USA; Department of Molecular Pharmacology and Experimental Therapeutics, Mayo Clinic; Rochester, MN, USA; Department of Molecular Pharmacology and Experimental Therapeutics, Mayo Clinic; Rochester, MN, USA; Department of Neuroscience, Mayo Clinic; Rochester, MN, USA; Department of Molecular Pharmacology and Experimental Therapeutics, Mayo Clinic; Rochester, MN, USA; Department of Molecular Pharmacology and Experimental Therapeutics, Mayo Clinic; Rochester, MN, USA; Epigenomics Program, Center for Individualized Medicine, Mayo Clinic; Rochester, MN, USA; Department of Laboratory Medicine and Pathology, Division of Experimental Pathology and Lab Medicine, Mayo Clinic; Rochester, MN, USA; Department of Molecular Pharmacology and Experimental Therapeutics, Mayo Clinic; Rochester, MN, USA; Department of Molecular Pharmacology and Experimental Therapeutics, Mayo Clinic; Rochester, MN, USA; Epigenomics Program, Center for Individualized Medicine, Mayo Clinic; Rochester, MN, USA; Department of Physiology and Biomedical Engineering, Mayo Clinic; Rochester, MN, USA; Division of Gastroenterology and Hepatology, Department of Medicine, Mayo Clinic; Rochester, MN, USA; Department of Molecular Pharmacology and Experimental Therapeutics, Mayo Clinic; Rochester, MN, USA

## Abstract

Understanding the function of non-coding genomic sequence variants represents a challenge for biomedicine. Many diseases are products of gene-by-environment interactions with complex mechanisms. This study addresses these themes by mechanistic characterization of non-coding variants that influence gene expression only after drug or hormone exposure. Using glucocorticoid signaling as a model system, we integrated genomic, transcriptomic, and epigenomic approaches to unravel mechanisms by which variant function could be revealed by hormones or drugs. Specifically, we identified *cis*-regulatory elements and 3D interactions underlying ligand-dependent associations between variants and gene expression. One-quarter of the glucocorticoid-modulated variants that we identified had already been associated with clinical phenotypes. However, their affected genes were ‘unmasked’ only after glucocorticoid exposure and often with function relevant to the disease phenotypes. These diseases involved glucocorticoids as risk factors or therapeutic agents and included autoimmunity, metabolic and mood disorders, osteoporosis and cancer. For example, we identified a novel breast cancer risk gene, *MAST4*, with expression that was repressed by glucocorticoids in cells carrying the risk genotype, repression that correlated with *MAST4* expression in breast cancer and treatment outcomes. These observations provide a mechanistic framework for understanding non-coding genetic variant-chemical environment interactions and their role in disease risk and drug response.

## INTRODUCTION

Many genetic sequence variants associated with human disease have been discovered, but the task of understanding the function of those variants remains challenging since most of them map to non-coding regions of the genome (1,2). One approach to address this challenge has been to associate these variants with gene expression, identifying so-called expression quantitative trait loci (eQTLs). Large-scale studies such as the Genotype-Tissue Expression (GTEx) Project have significantly improved our understanding of steady-state eQTLs across different tissues (3). However, it is increasingly understood that eQTLs can be dynamic – that is, sequence variants become associated with variation in gene expression only after specific environmental stimulus (4). For example, variants which appear to be ‘silent’ at baseline become functional after pathogen invasion (5). Our group (6–8) and others (9,10) have observed a series of uniquely ‘pharmacologic’ eQTLs, hereafter referred to as ‘pharmacogenomic (PGx)-eQTLs’, for which eQTL behavior is elicited or significantly amplified in the presence of a drug or hormone. These dynamic eQTLs not only explain novel functions of non-coding variants but also provide valuable insight into molecular mechanisms underlying gene-by-environment interactions, interactions which could play important roles in complex disease pathophysiology (11).

**Figure 1.**
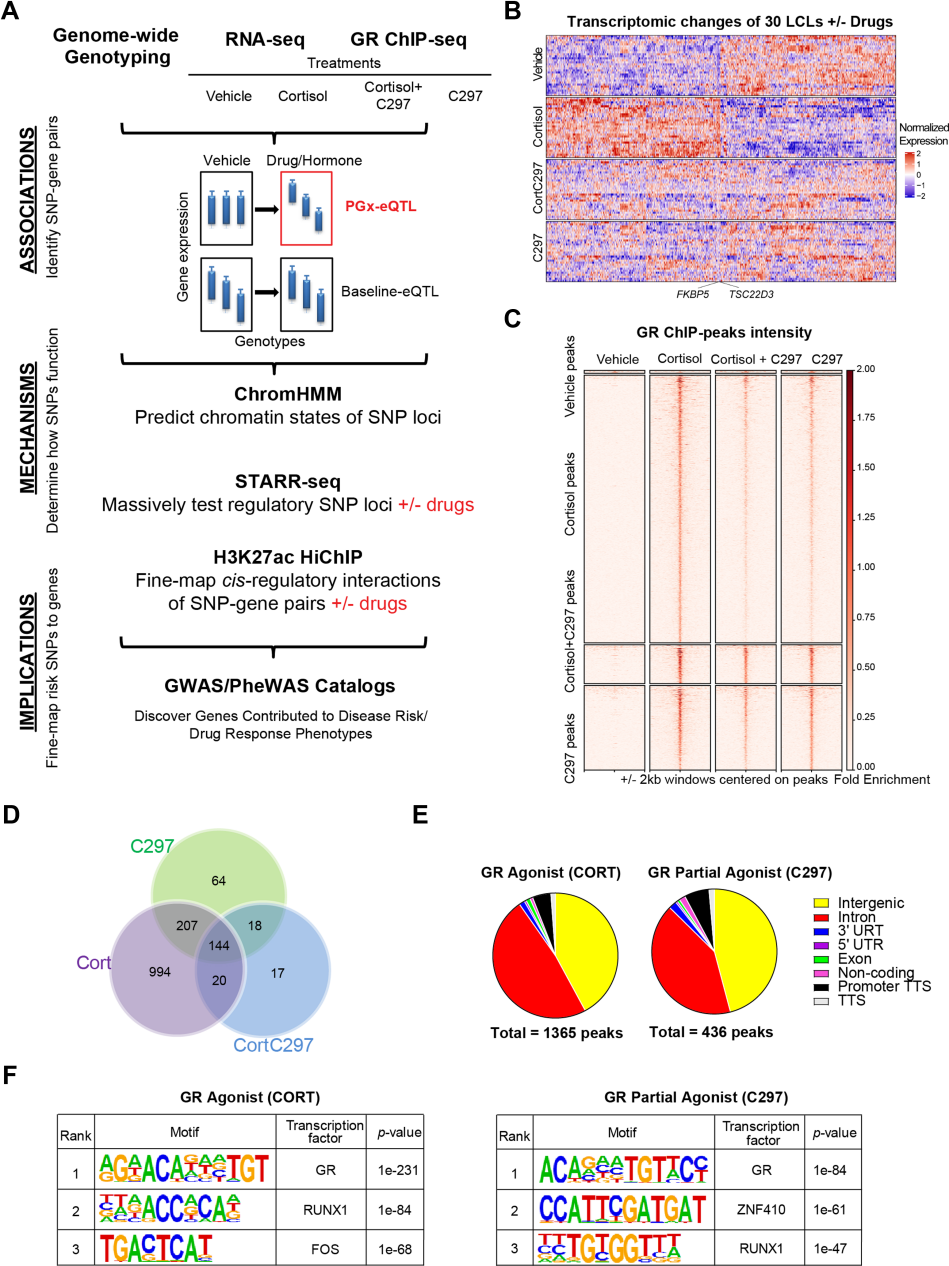
**Conceptual framework for the study**. (**A**) Experimental design used in this study. ChromHMM is a software used to annotate chromatin states from epigenomic data. STARR-seq stands for self-transcribing active regulatory region sequencing. Cortisol is a GR agonist, C297 is a GR modulator which acts as an antagonist when administered together with cortisol and a partial agonist by itself. (**B**) Heatmap of differentially expressed genes (FDR < 0.05) after four treatment conditions across 30 LCLs showing drug-dependent patterns of gene regulation. Rows represent individual cell lines, and columns represent individual genes. (**C**) GR-targeted ChIP peak intensity after normalization to input across the 4 treatment conditions used shows drug-dependent patterns similar to those of RNA-seq. (**D**) Overlap of GR-targeted ChIP-peaks across 4 treatment conditions shows that C297 also acts as a partial agonist since the majority of C297-induced GR peaks overlapped with cortisol-induced peaks. (**E**) The distribution of GR-targeted ChIP peaks shows major enrichment in intronic and intergenic regions. (**F**) The top three *de novo* motifs identified by HOMER for GR-targeted ChIP-seq peaks after cortisol or C297 treatment demonstrates peak specificity for GR.

While dynamic eQTLs have been identified in several conditions, the mechanisms by which the impact of genetic variants on gene expression is ‘unmasked’ by environmental stimuli remain largely unknown. This study was designed to interrogate mechanistically the interaction between non-coding genetic variants and glucocorticoids, important agents used to treat a wide range of disease but at times causing serious side-effects (8,12–17), and their role in disease risk by integrating a series of pharmacogenomic and pharmacoepigenomic datasets. Specifically, our study design (Figure 1A) began with the identification of PGx-eQTLs using genome-wide single-nucleotide polymorphisms (SNPs), and RNA-seq and GR-targeted ChIP-seq before and after exposure to glucocorticoids in immortalized human lymphoblastoid cell lines (LCLs) of differing genomic backgrounds. To validate drug-dependent effects, we treated these cells with cortisol, a GR agonist, and the drug CORT108297 (C297), a selective GR modulator which, in our studies, displayed antagonist properties when administered with cortisol and partial agonist activity by itself. Cortisol and its sister compounds are used routinely in the clinic to treat immunity-related diseases (13,18,19), while C297 is a selective GR modulator currently being tested for the treatment of post-traumatic stress disorder (trial NCT04452500) and Alzheimer's disease (trial NCT04601038). We then applied a series of epigenomic techniques including integrative chromatin state prediction (ChromHMM), a massively parallel reporter gene assay (STARR-seq) +/− drugs, and 3D chromatin conformation capture targeting *cis*-regulatory elements bound by the active enhancer- and promoter-associated histone mark acetylated histone H3 lysine 27 (H3K27ac HiChIP) +/− drugs. The integration of these datasets made it possible for us to determine underlying mechanism(s) and to generate additional evidence for associations underlying the observed PGx-eQTLs. Finally, we could then identify which of the discovered PGx-eQTLs might help to explain disease risk mechanisms by overlapping our SNPs with significant signals from publicly available genome-wide and phenome-wide association studies (GWAS and PheWAS).

## MATERIALS AND METHODS

### Generation of SNP data

287 lymphoblastoid cell lines (LCLs) were obtained from the Coriell Institute. DNA from these 287 LCLs was genotyped with the Affymetrix Human SNP Array 6.0 at the Coriell Institute, and with the Illumina HumanHap550K and HumanExon510S-Duo Bead Chips in our laboratory. The genotype data were deposited in the National Center for Biotechnology Information Gene Expression Omnibus (GEO accession: GSE23120) (20).

### Cell culture and drug conditions

LCLs were cultured in RPMI 1640 supplemented with 15% FBS and 1% penicillin/streptomycin. A549 (ATCC^®^) cells were cultured in F12-K supplemented with 10% FBS and 1% penicillin/streptomycin. MCF-7 (ATCC^®^) cells were cultured in EMEM supplemented with 10% FBS and 1% penicillin/streptomycin. HCC1954 (ATCC^®^) cells were cultured in RMPI 1640 supplemented with 10% FBS and 1% penicillin/streptomycin. Cell culture conditions for these cell lines were 37°C and 5% CO2. MDA-MB-231 (ATCC^®^) cells were cultured in L-15 supplemented with 10% FBS. Cell culture condition for MDA-MB-231 were 37°C without CO_2_.

Before glucocorticoid treatment experiments, all cells were grown in 5% charcoal-stripped media for 48 h. Drug conditions and dosages were as follow: (i) Vehicle (dimethyl sulfoxide (DMSO) 0.1% and ethanol 0.1%), (ii) hydrocortisone 100nM (Sigma Aldrich, dissolved in ethanol) plus DMSO 0.1%, (iii) CORT108297 100nM (C297, AchemBlock, dissolved in DMSO) plus ethanol 0.1%, (iv) hydrocortisone 100 nM plus C297 100 nM (hydrocortisone is the name for the hormone cortisol when supplied as a medication). Physiologically relevant dosages (<1000 nM) and time points were optimized based on strongest induction of mRNA expression of GR-responsive canonical genes *FKBP5* and *TSC22D3* before saturation point.

### RNA-sequencing experiments

Thirty steroid-starved LCLs with similar expression of GR selected from the 300-LCL panel (Supplementary Table S1) were subjected to the four drug exposure conditions in serum-free media for 9 h. After treatment, cells were pelleted, and total RNA was extracted with the RNAeasy Mini Kit per manufacturer's instruction (Qiagen). DNase on-column treatment was performed with the DNase I set (Zymo). RNA integrity number for all samples was 10. RNA-seq libraries were prepared with the TruSeq RNA Library Prep Kit v2 (Illumina). Paired-end sequencing 2 × 100 bp was conducted on an Illumina HiSeq 4000 with a sequencing depth of ∼25 million paired-end reads per sample. Raw RNA sequencing reads were aligned to the human genome GRCh37 (hg19) using STAR (21). Raw counts were generated with the Python package ‘HTseq’ (22) and normalized using conditional quantile normalization method (CQN). Only genes that passed normalized counts of 32 in at least 15 cell lines and one drug condition were retained. Downstream differential expression analysis was conducted with the R package ‘EdgeR’ (23) using a quasi-likelihood model.

### Glucocorticoid-targeted chromatin immunoprecipitation (ChIP)-sequencing experiment

Steroid-starved GM17261 cells were subjected to the four drug exposure conditions in serum-free media for 1 h. Twenty million cells for each condition were then cross-linked with 1% methanol-free formaldehyde (Thermo Fisher) for 10 min at room temperature. The reaction was then stopped with 125 mM glycine for 5 min at room temperature and the pellets were frozen at −80°C prior to extraction. Chromatin preparation for ChIP-seq was performed as described by Zhong *et al.* (24). A cocktail of antibodies against the glucocorticoid receptor was added to chromatin input for immunoprecipitation: 2 μg of ab3579 (Abcam, lot GR3222141-5, discontinued) and 0.49 μg of 12041S (Cell Signaling Technology, lot 3) per 20 million cells. A separate set of ChIP-seq assays to validate reproducible peaks was conducted with 10 μL of 12041S (Cell Signaling Technology, lot 3) per 20 million cells. Please refer to Supplementary Methods for step-by-step details of the protocol. After library preparation, paired-end sequencing 2 × 50 bp was performed on an Illumina HiSeq 4000 with sequencing depth of ∼12.5 million paired-end reads per sample. Raw sequencing reads were processed and analyzed using the HiChIP pipeline (25) to obtain integrative genomics viewer files and a list of peaks (FDR < 0.01). Overlapped peaks between two datasets were evaluated with the R package The ChIPpeakAnno (26), and the correlation of scores for overlapped peaks was evaluated with Pearson correlation.

### Massively parallel reporter assay (STARR-seq) experiments

For step-by-step details, please refer to Supplementary Methods.

Human STARR-seq-ORI vector was obtained from Addgene (plasmid #99296). For inserts, we amplified all identified PGx-eQTL peaks extended by ±500 bp using genomic DNA extracted from the 30 LCLs (Supplementary Table S1) (for primers sequences and specific PCR conditions, refer to Supplementary Data 4). We included in the library a GR-induced peak within a strong enhancer region near the promoter of *FKBP5*, extended by ±500 bp, as a positive control. Assembled DNA products were then chemically transformed into NEB^®^ 5-alpha competent *E. coli*, expanded and harvested. Plasmids were extracted with the HiSpeed Plasmid Maxi Kit (Qiagen). The quality of the inserted DNA loci (input library) was checked by amplification of the loci using universal primers, which were then sequenced on a Hiseq4000 (Illumina) with 150-bp paired-end sequencing.

LCLs and A549 were maintained at viability of > 90% before transfection. For LCLs, a total of 600 ug plasmids were transfected into 240 million cells. For A549, a total of 450 ug plasmids were transfected into 225 million cells. After electroporation, cells were treated with drugs (hydrocortisone, C297 or vehicle) in 5% CS media for 9 h. Total RNA was then harvested with the RNA Maxiprep kit per manufacturer's instructions (Qiagen).

Messenger RNA was isolated using the Dynabeads Oligo (dT)_25_ (Invitrogen), followed by TURBO™ DNase digestion. We then conducted first-strand cDNA synthesis with the SuperScript III Reverse Transcript kit (Invitrogen) using a reporter transcript-specific primer and no more than 500ng mRNA per reaction. cDNA pool was then treated with RNAseA and purified with AMPure XP beads (Beckman). We then conducted junction PCR, the products of which were purified, sheared, adapter-ligated with NEBNext® Ultra™ DNA Library Prep Kit, and sequenced on an Illumina Hiseq 4000 with pair-end mode 2 × 150 bp.

Adapter sequences and reads aligned to universal plasmid sequences were trimmed out, and the trimmed reads were then aligned to GRCh37 (hg19) with BWA (27) mem using default parameters. Only reads with MAPQ ≥30 were kept for further analyses. Samtools (28) were used to convert sam files to bam files and to sort the bam files. Read counts for all SNPs sequenced within each PGx locus were then called with bcftools mpileup. Read counts of loci expression were called with the Python Package ‘HTseq’ (22). Read counts for each sample were then normalized by library size and were log (CPM) transformed. Indels, multi-allelic variants, and variants with counts less than 20 were removed. All differential analyses were conducted with the R package ‘EdgeR’ (23). For SNP-dependent loci analysis, the difference between the proportions of alternative alleles in vehicle- and drug-treated samples were evaluated using two-tailed Fisher's exact test. Statistical significance was defined as FDR < 0.05, and % alternative allele difference (Alt_drug_/Total_drug_ – Alt_vehicle_/Total_vehicle_) > 10%. Visualization of STARR-seq data was conducted with the ‘EnhancedVolcano’ R package.

### Chromatin conformation capture of enhancer–enhancer/promoter interactions surrounding H3K27ac mark (HiChIP)

#### Publicly available data (baseline)

H3K27ac HiChIP data in GM12878 were downloaded from Mumbach *et al.* (29) and analyzed with the MAPS pipeline (30) using default parameters and known H3K27ac ChIP-seq peaks from ENCODE as anchors. All loops were called at 5kb bin size and were defined as having at least one end overlapping with a H3K27ac peak. PGx-eQTL SNPs were then overlapped with loop data to identify which SNP loci had H3K27ac-loop contact with eQTL genes using the R package ‘GenomicRanges’ (31). H3K27ac HiChIP data in MDA-MB-231 cells were downloaded from Cho *et al.*, 2018 (32) and analyzed with the MAPS pipeline (29) using similar default parameters and H3K27ac ChIP-seq peaks were called directly from HiChIP sequencing.

#### Data generated in this study (before and after drug treatment)

Steroid-starved GM17261 cells was subjected to vehicle (0.01% EtOH) and 100 nM of cortisol in serum-free media for 9 h. After treatment, a portion of the cells was collected for RNA extraction and qRT-PCR to validate the effect of drug treatment on gene expression. Fifteen million cells per condition were fixed by 2% methanol-stabilized formaldehyde (Fisher Scientific) at room temperature for 10 min. The reaction was then stopped with 125 mM glycine for 5 min at room temperature. Cell pellets were snap-frozen in liquid nitrogen and sent to Arima Genomics (San Diego, CA, USA) for H3K27ac HiChIP library preparation. After passing quality control by shallow sequencing, the libraries were sequenced on an Illumina NovaSeq, yielding 500–800M pair-end reads per sample. HiChIP data were analyzed using the MAPS pipeline (30) using default parameters. Loops were called with a bin size of 5 kb, maximum loop distance of 2000 kb, and false discovery rate (FDR) of <0.01. Valid interacting pairs were defined as having at least one end overlapping with an H3K27ac peak. Two downstream analyses were conducted: (i) to identify loops before and after drug treatment which connected cortisol-dependent DEGs and cortisol ChIP-seq, (ii) to identify loops before and after drug treatment which connected PGx SNPs and eQTL genes directly or through a common contact using the R package ‘GenomicRanges’ (31) and bedtools (33). All genes were extended for 2000 bp at the 5′ end (using Ensembl hg19 gene annotation) to include promoter regions.

### Pharmacogenomic-eQTL analysis

The number of SNP-gene pairs included in the eQTL analysis was narrowed down to leverage statistical power. Specifically, 1.3 million genotyped SNPs were filtered based on Hardy-Weinberg Equilibrium (*P* > 0.001), genotyping call rates of more than 95%, and a minor allelic frequency of 0.18 to retain the probability of at least 1 cell line with the minor allelic genotype, resulting in 808 875 SNPs. We then identified SNPs within *cis* distances of ±200 kb from genes, resulting in 433 272 SNPs. RNA-seq raw reads were normalized using conditional quantile normalization. All genes that mapped to sex chromosomes were excluded. Only genes with raw counts of ≥32 in at least one treatment and half of the LCLs were considered for eQTL analysis. All treatment conditions were normalized to vehicle by genotypes to remove genotype effects at baseline and to adjust for cell line-dependent factors such as sex and age. Analysis of eQTLs for normalized expression of drug/vehicle expression was conducted with the R package ‘Matrix eQTL’ (34) using ANOVA model. Identified *P*-values of SNP-gene pairs after drug treatment were then compared with those obtained from analysis of 174 LCLs at baseline in the GTEx database with a significance cutoff of 0.05. PGx-eQTLs were also evaluated to determine whether the associations were lost after the antagonist treatment, demonstrating drug-dependent properties. SNPs in tight linkage disequilibrium (LD) (*r*^2^ > 0.4 and 0.8) with the identified PGx SNPs were investigated with regard to their potential to create/disrupt a known GR binding motif using the HaploReg v4.0 database. For significant SNP-gene pairs, we also conducted analyses of all SNPs within 200 kb *cis* distance to compare the statistical significance of SNPs outside and inside GR binding sites, the results of which were plotted using the R package ‘Circlize’ (35). For selected examples depicted in the manuscript, post-hoc ANOVA Tukey's test was used to evaluate significance for differences between homozygous wildtype and variant genotypes with GraphPad Prism 8.0.

PGx-eQTL SNPs were then overlapped with those documented in GWAS Catalog, UKBiobank data, or FinGen study data to identify SNPs that have been associated with a clinical phenotype and whether the phenotype might fit with current knowledge about GR function. The *P-*values cutoff for GWAS and PheWAS were the same as those defined by the databases.

### ChromHMM analysis

Epigenomic datasets on GM12878 (LCL, Caucasian) were downloaded from the ENCODE project (36,37) for the following epigenetic marks/regulators: H3K4me1, H3K4me3, H3K27ac, H3K9me3, H3K27me3, H3K36me3, H3K4me2, H3K9ac, H4K20me1, H3K79me2, POLR2A, H2AFZ, DNase hypersensitive sites, CTCF, and EP300 (38) (see Supplemetary Data S3 for samples ID). BAM files of the LCL epigenetic marks/regulators, together with the BAM files of GR ChIP-seq after cortisol and C297 treatment, were binarized and segmented into 15, 18 or 25 chromatin states with default parameters using ChromHMM (39). The 25-state value was adopted for its ability to capture as many states as possible without redundancy. These states were then annotated based on combinatorial and spatial patterns of chromatin marks (40). Genomic coordinates of the chromatin states were converted from GRCh38/hg38 to GRCh19/hg19 using UCSC liftOver. Enrichment of PGx-eQTL SNPs in each of the 25 states was then analyzed using bedtools (33).

### Functional validation of selected disease-associated PGx-eQTL experiments

#### Luciferase reporter gene assays

To validate selected STARR-seq signals with clinical significance, we conducted luciferase reporter gene assays using the STARR-seq luciferase validation vector (Addgene #9927). The plasmid was digested with restriction enzymes BamHI-HF® (Cat#: R3136S, NEB) and SspI-HF® (Cat#: R3132S, NEB). We used Gibson Assembly cloning approach, the conditions of which were similar to those described for STARR-seq experiments, to create luciferase constructs. Before being cloned in the plasmid, the PGx loci were created by PCR amplification of the genomic DNA from LCLs with known genotypes for the SNP of interest. Primers were designed with NEBuilder Assembly Tool 2.0 and NEBcutter v3.0. Please refer to Supplementary Data S4 for specific primer sequences of each locus. After confirmation of the genotype by Sanger sequencing, reporter gene constructs containing wildtype or variant SNP genotypes were transfected into cells. A pRL-TK vector that expresses Renilla luciferase (Cat#: E2241, Promega) was co-transfected as an internal control. For LCLs, 200ng of Renilla plasmid was co-transfected with 800ng of Luciferase construct into 1 million cells using the Lonza SF Cell Line 4D-Nucleofector™ X Kit L (Cat#: V4XC-2012, Lonza). For MDA-MB-231 breast cancer cells, 100 ng of Renilla plasmid was co-transfected with 400ng of Luciferase construct into 0.5 million cells using the TransfeX™ Transfection Reagent (Cat#: ACS-4005, ATCC). After 24 h of glucocorticoid treatment, the cells were lysed, and luciferase activity was determined using the Dual-Luciferase® Reporter Assay System kit (Cat#: E2241, Promega). For every condition, three biological replicates were included. Statistical significance across genotype groups was determined with student's two-tailed *t*-test using GraphPad Prism.

#### Glucocorticoid-targeted ChIP-qPCR

For step-by-step details, please refer to Supplementary Methods.

To test GR activity for the rs1697139-*MAST4* PGx-eQTL locus in a triple negative breast cancer cell line, MDA-MB-231 cells were steroid-starved for 48 h and subjected to vehicle and 100nM dexamethasone treatment for 1.5 h (dexamethasone instead of cortisol was used to avoid cortisol effect on the mineralocorticoid receptor since, unlike LCLs, MDA-MB-231 expressed the mineralocorticoid receptor at reasonable levels). To test SNP-dependent GR activity for rs12834655-*RUNX1* PGx-eQTL locus, GM17215 (rs12834655 genotype AA) and GM17293 (rs12834655 genotype GG) cells were steroid starved for 48 h and subjected to vehicle and 100nM cortisol treatment for 1.5 h. Chromatin preparation was conducted similarly to that during ChIP-seq experiment. Each qPCR reaction was then conducted with the *Power* SYBR™ Green PCR Master Mix (Thermo Fisher, Cat# 4367659) in triplicates. The Ct numbers of ChIP DNA were then normalized to Ct numbers of input DNA, and then normalized to Ct numbers of IgG. Data graphs were plotted using Prism (GraphPad Software). Statistical comparisons between genotypes were made using two-tailed Student's *t*-test.

#### qRT-PCR of MAST4 in breast cancer cell lines after glucocorticoids treatment

To test the effect of GR signaling on *MAST4*, a gene that was a PGx-eQTL with the breast-cancer-associated rs1697139 SNP locus, 3 cell lines with reasonable expression of GR (41) and MAST4 representing three common subtypes of breast cancer were subjected to treatment with 100nM Dexamethasone (Sigma, water soluble) for 2hrs, 6hrs, and 24hrs. Total RNA was extracted with Direct-zol RNA Miniprep (Zymo, Cat# R2052) per the manufacturer's instructions. mRNA levels for *MAST4*, *GADPH*, and *FKBP5* were determined by qRT-PCR using the Power SYBR™ Green RNA-to-CT™ 1-Step Kit (Applied Biosystems Inc.). 100ng of total RNA was used for each reaction. Because MDA-MB-231 showed the most dramatic repression of *MAST4* after dexamethasone exposure, a dose-dependent 6-hour treatment with dexamethasone at 0nM, 1nM, 10nM, and 100nM was conducted on MDA-MB-231 cells, followed by qRT-PCR to confirm the drug effect. qRT-PCR was run with three technical replicates. Analysis of qRT-PCR was conducted using the *2*^−ΔΔ^*^CT^* method. Data graphs were then plotted using Prism (GraphPad Software). Statistical comparisons between genotypes were made using two-tailed Student's t-test.

#### CRISPR-Cas9 experiments targeting enhancer region in breast cancer cell line

Guide RNAs (gRNAs) were designed to cut 1–2 kb surrounding the SNP region with the CRISPR Targets Track on UCSC Genome Browser. Those gRNAs needed to pass a specificity score of 70, and an efficiency score of 80%. Sequence of single gRNA sg1 was GCGATCCAATCTCACAGGGG (77 specificity score, 94% efficiency) and of sg2 was GTTTAAACCAACTAGACCCC (90 specificity score, 84% efficiency). Both sg1 and sg2 were synthesized by the Integrated DNA Technology (IDT) and were then assembled *in vitro* with tracrRNA and Cas9 Nuclease (Cat#: 1081058, IDT) separately to form the ribonucleoprotein (RNP) complex according to the manufacturer's instruction. Delivery of the Alt-R CRISPR-Cas9 RNP complex into MDA-MB-231 cells was conducted with the SE Cell Line 4D-Nucleofector Kit X (Cat# V4XC-1032, Lonza) according to the manufacturer's instruction in combination with the Alt-R® Cas9 Electroporation Enhancer (Cat#: 1075915, IDT). The result of CRISPR/Cas9 editing was evaluated with PCR and gel electrophoresis. Specifically, cells were lysed with the DNAzol® Direct (DN 131, Molecular Research Center) and the lysates were directly used as PCR templates. The sequences of primers for amplification of the edited sites were TGTTGTCAGGGCCTTTGAGA (forward) and CGTCACCAGGATAGCAAGCT (reverse). The PCR was conducted with the KAPA HiFi HotStart ReadyMix PCR Kit (Cat#: KK2601, Roche) and the condition for PCR was as follow: 95°C for 3 min, followed by 25 cycles of 98°C for 20 s, 65°C for 1.5 min, and 72°C for 60 s. PCR product was visualized in 1.0% agarose gel after electrophoresis. The expected size of PCR products was 1921bp for wildtype genotype and 862bp for KO genotype. After conducting experiments with edited bulk cells, we proceeded to select for single colonies of homozygous KO. Please see Supplementary Methods for details. Out of a total of 192 colonies were screened, two colonies were homozygous KO. KO colonies were expanded and re-genotyped before drug treatment experiment. Treatment experiments were conducted similarly as described above, with 100 nM of dexamethasone for 6 h.

#### MAST4 expression in patient tumor samples

To assess *MAST4* expression in tumor samples from different subtypes of breast cancer as compared to normal tissue, data were downloaded from The Cancer Genome Atlas (TCGA). Two-tailed unpaired Student's t-test was used to compare *MAST4* expression between normal and cancer tissues. To assess *MAST4* expression and its relationship to relapse free survival (RFS) of breast cancer patients of all subtypes who underwent endocrine or chemotherapy, Kaplan-Meier plots were generated using KM plotter (42) and its associated breast cancer database (43). To address the concern that the association of *MAST4* expression with lower RFS was driven by its most significant repression in TNBC, which usually had a worse survival rate that other subtypes, Kaplan–Meier plots were also stratified by subtype, and *MAST4* low expression was predictive of lower RFS in other subtypes just as was the case with TNBC.

## RESULTS

### Genome-wide discovery of glucocorticoid-modulated PGx-eQTLs in human LCLs

A total of 120 transcriptomic profiles were obtained under four drug conditions—cortisol, C297, both together and vehicle control for 30 LCLs (Supplementary Table S1), as well as genome-wide GR-binding profiles under the same drug exposure conditions for a randomly selected LCL. The global changes of these profiles were characterized to ensure that the responses were glucocorticoid-specific. Because LCLs do not express or express only a very low level of mineralocorticoid receptor (3), another endogenous receptor to which cortisol can bind, the treatment effects that we observed were GR-specific. We found that cortisol regulated the expression of 1361 genes across these 30 cells (FDR < 0.05) including GR canonical genes such as *FKBP5* and *TSC22D3*, whereas only 26 genes remained differentially expressed when C297 was added in combination with cortisol. Furthermore, C297 also acted as a partial agonist since, when tested alone, it upregulated a group of GR-target genes but to a lesser extent than cortisol, genes that included *FKBP5* and *TSC22D3* (see Figure 1B for a visualization of drug-dependent transcriptomic patterns and Supplementary Data 1 for a complete list of differentially expressed genes).

Similar drug-dependent patterns were observed in the GR-targeted ChIP-seq assays. Specifically, cortisol induced 1365 peaks and C297 induced 436 peaks at a 0.01 FDR threshold. These values were reduced to 200 peaks when the two drugs antagonized each other (Figure 1C, D). Approximately 85% of the C297-peaks overlapped with cortisol-peaks, suggesting that C297 is a partial agonist, as shown in the RNA-seq data (Figure 1D). In terms of distribution, GR peaks induced by either drug mapped predominately within intergenic and intronic regions, consistent with previous knowledge of GR function (44) (Figure 1E). We also demonstrated that these peaks were of high specificity, since *de novo* motif analysis showed that the peaks for both cortisol and C297 were most highly enriched in GR binding motifs (*P* = 10^−231^ for Cortisol, *P* = 10^−84^ for C297) (Figure 1F). To evaluate peak reproducibility, we conducted an independent replication of the GR-targeted ChIP-seq using different GR antibodies. We observed a similar number of peaks and achieved a high reproducible rate of 70% replicated peaks at a stringent FDR threshold of 0.01 (see Supplementary Figure S1a). There was a strong correlation of peak score between the replicates for the reproducible ChIP-seq peaks (see Supplementary Figure S1c). We also observed a strong drug-dependent peak pattern similar to that observed in the original dataset (see Supplementary Figure S1b). After demonstrating that glucocorticoids displayed robust effects on RNA-seq and ChIP-seq, we set out to identify drug exposure-dependent eQTLs.

**Figure 2.**
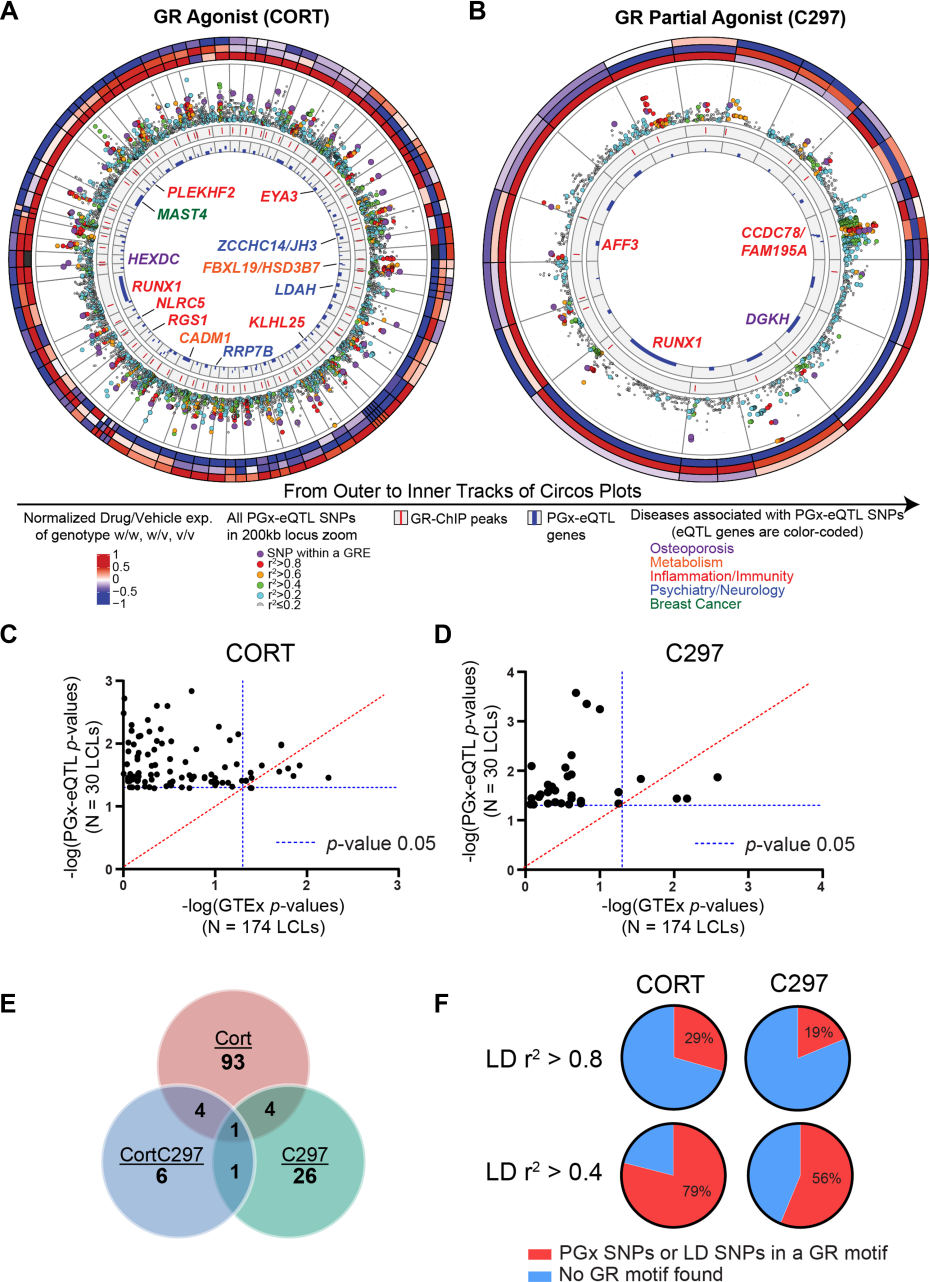
**Discovery of GR-modulated PGx-eQTLs in human LCLs**. (**A**, **B**) Circos plots depicting the GR-dependent PGx-eQTLs identified in 30 LCLs with results for (**A**) cortisol (CORT) and (**B**) the partial agonist (C297). The three outermost circles represent relative gene expression values (drug/vehicle) for each genotype, with each column depicting a PGx SNP-gene pair. The inner tracks are explained in the figure. The track with locus zoom plots shows that SNPs within a GR binding site were generally more significantly associated with eQTL genes than other SNPs within the 200kb window of a gene. (**C**, **D**) *P*-Values for eQTL analyses from our study using 30 LCLs (Y axis) vs *P*-Values for eQTL analyses using 174 GTEx LCL samples. The majority of the PGx-eQTLs identified in the present study were not significant in GTEx even with larger sample sizes. (**E**) The number of SNP-gene pairs identified for each drug condition and their overlap across conditions. The majority of PGx-eQTL SNP-gene pairs after cortisol or C297 treatment no longer existed after antagonism (CortC297) was introduced. (**F**) Percentages of identified PGx-eQTL SNPs that mapped within a known GR binding motif or in tight LD with SNPs within a GR motif according to HaploReg V4.0 database.

To account for the limited power for eQTL analysis, we narrowed the number of SNP-gene pairs by first selecting only *cis*-SNPs and genes that passed stringent quality control criteria (see Methods). We then selected SNPs that mapped within or near (±500 bp) GR binding sites by overlapping SNPs with the GR-targeted ChIP-seq data to focus on those most likely to interfere directly with GR signaling, acknowledging that relevant SNPs with different possible mechanisms of action (e.g. SNPs within binding sites for downstream transcription factors regulated by GR) would be missed. As a result, a total of 1838 SNP-gene pairs for cortisol and 572 for C297 were included in the eQTL analysis. We identified 102 cortisol-dependent and 32 C297-dependent *cis* PGx-eQTL SNP-gene pairs, of which 5 were shared between the 2 conditions (Figure 2A, B, E, Supplementary Figure S2), the majority of which lost their cortisol-dependent eQTL status when exposed to cortisol and C297, an antagonist, thus demonstrating, in a compelling fashion, the drug-dependent properties of this type of eQTL (Figure 2E). Furthermore, these PGx-eQTLs were not significant baseline eQTLs based on data from 174 LCLs deposited in the GTEx database (Figure 2C-D). We observed that the SNPs themselves either mapped within known GR binding motifs, were in tight linkage disequilibrium with SNPs within GR motifs, or were distant from GR motifs (Figure 2F; see Supplementary Data 2 for details for each SNP). However, in all cases, they could still influence GR-dependent transcriptional activity, as later confirmed by massively parallel reporter gene assay.

### Glucocorticoid-modulated PGx-eQTLs most often mapped to enhancers with looping properties

**Figure 3.**
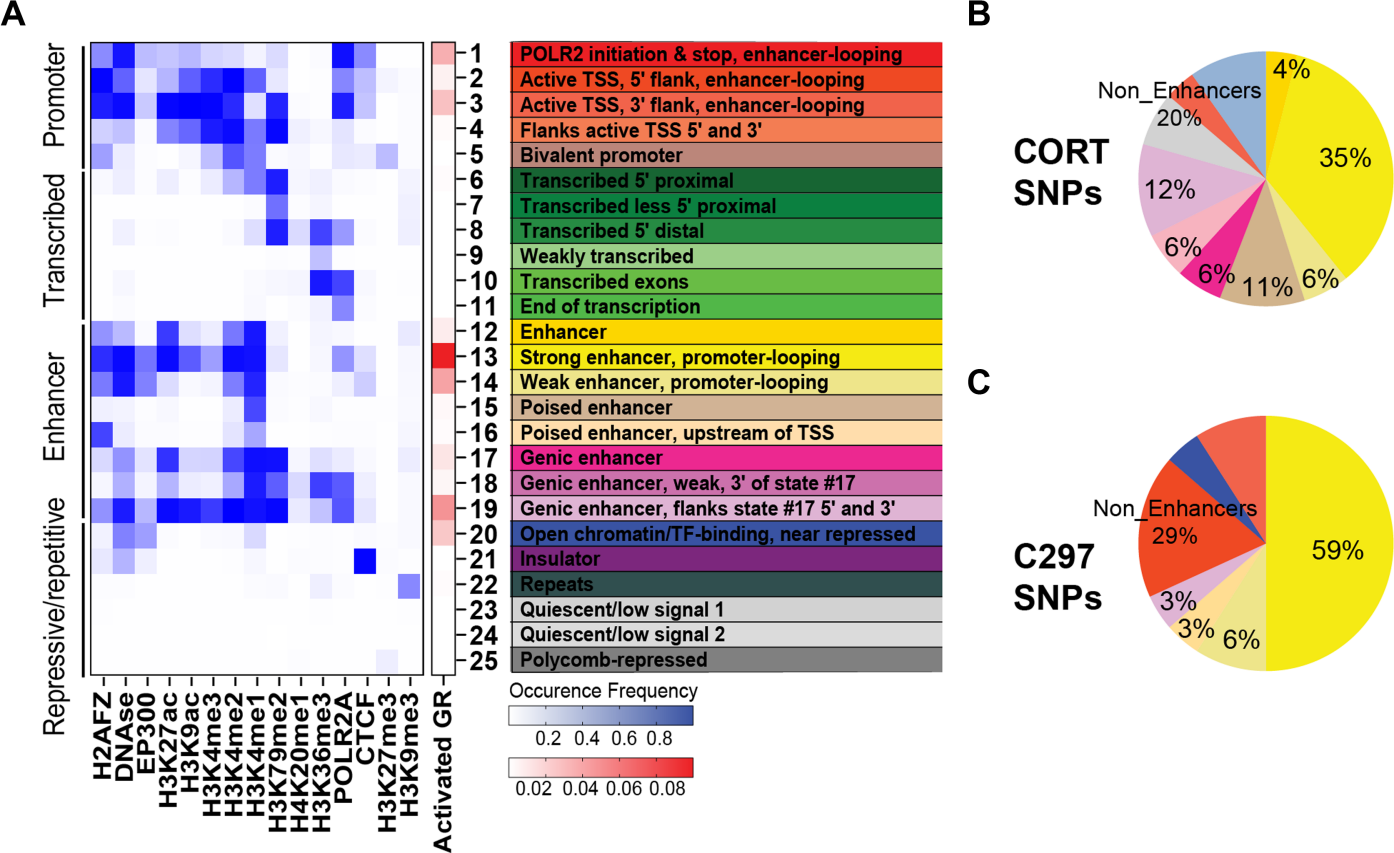
**Prediction of chromatin states for GR-modulated PGx-eQTLs**. (**A**) 25 LCL chromatin states predicted from the occupancy of 15 epigenetic marks for the reference LCL from ENCODE and the enrichment of GR peaks within each state. Columns represent epigenetic marks. Rows represent the co-occurrence probability of epigenetic marks within a state. (**B, C**) Distribution of GR-dependent PGx-eQTL SNPs among different chromatin states, which have been color coded as in (**A**).

Using ChromHMM, a software that models chromatin signatures with a multivariate Hidden Markov Model to annotate the putative regulatory function of the noncoding genome using epigenomic information (39), we integrated 15 LCL epigenomic datasets from the Encyclopedia of DNA Elements (ENCODE) portal (36) with our GR-targeted ChIP-seq data (see Supplementary Data 3 for information on datasets). The 25-predicted chromatin states obtained were then annotated based on combinatorial and spatial patterns of chromatin marks (40) (Figure 3A, Supplementary Figure S3, Supplementary Table S2). These states could be categorized into four broad categories: (i) Promoter, (ii) Enhancer, (iii) Transcribed and (iv) Repressive/Repetitive/Unknown. Overlapping of GR-dependent PGx-eQTLs with these chromatin states demonstrated that PGx-eQTLs were enriched in a variety of states but predominantly in enhancers, with the primary site of enrichment being long-range enhancers that displayed promoter-looping properties (Figure 3B, C, Supplementary Table S3; see Supplementary Data 2 for details on each SNP). Specifically, 81% of cortisol-modulated PGx-eQTLs mapped to enhancer-related states and 41% mapped to enhancers with predicted looping properties. For C297-modulated PGx-eQTLs, 68% mapped to enhancer-related states and 65% mapped to enhancers with predicted looping properties.

### Allele-dependent and drug-dependent properties of enhancer PGx-eQTLs were replicated across cell lines

**Figure 4.**
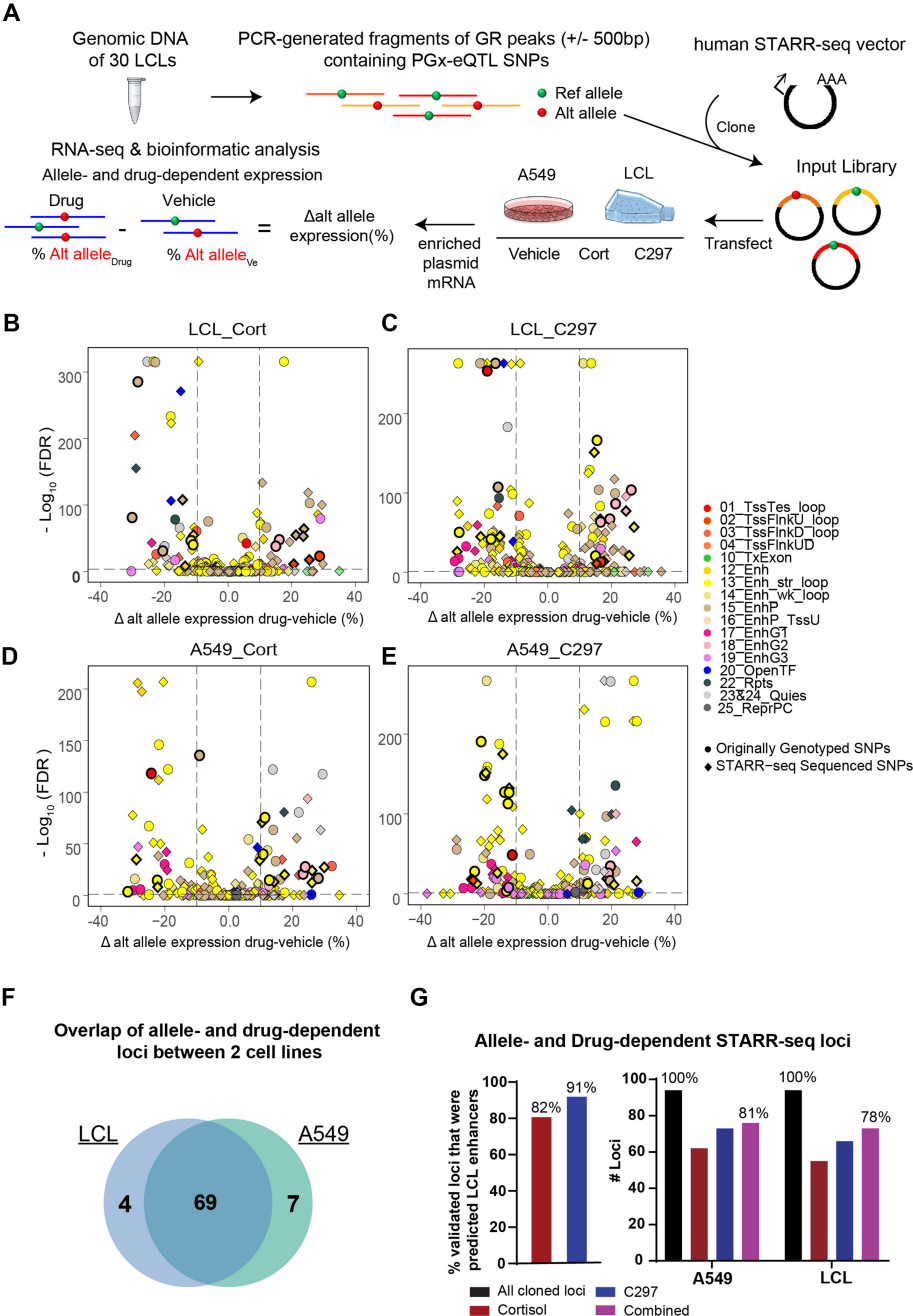
**Testing allele- and drug-dependent effects of PGx-eQTLs with STARR-seq, a massively parallel reporter enhancer assay**. (**A**) Experimental workflow for STARR-seq. (**B–E**) Volcano plots showing PGx-eQTL loci where SNP-dependent and GR-dependent activities were detected. The Y axis represents –log10 of FDR from Fisher's Exact Test, the X axis represents change of alternative allele percentages after drug treatment. Circles represent originally genotyped SNPs, and squares represent SNPs sequenced in each STARR-seq locus. Each SNP was color coded by the chromatin state in which they resided. All loci that were later found to be associated with diseases that achieved statistical significance for SNP-dependent and GR-dependent transcriptional analysis are bolded in black. (**F**) High consistency between the two cell lines, LCL and A549, used in the STARR-seq assay in terms of loci that displayed allele- and drug-dependent properties. (**G**) STARR-seq results validated allele- and drug-dependent enhancer activities of identified PGx-eQTL loci.

Because the majority of the PGx-eQTL SNPs that we had identified mapped to enhancer regions, we next performed STARR-seq, a massively parallel reporter gene assay that can capture enhancer activity in a high-throughput fashion (45), to verify the effect of these SNPs and drugs on transcriptional activity associated with the identified PGx-eQTLs (see Figure 4A for a diagram of the experimental workflow). We first cloned PCR-generated PGx-eQTL locus fragments from the pool of genomic DNA from our 30 LCLs into the human STARR-seq vector. Those fragments covered the GR binding peaks (±500 bp) that contained the identified PGx-eQTL SNPs. We included a glucocorticoid-induced GR peak in the library that mapped to a strong enhancer region near the *FKBP5* promoter as a positive control (Supplementary Figure S4a). We then transfected the STARR-seq libraries into LCLs and A549 cells, a lung cancer cell line in which GR genomic regulation has been studied extensively (44), exposed the cells to cortisol or C297, extracted mRNA and enriched the targeted sequences by RT-PCR. As expected, the transcribed products showed a size of around 1300bp (Supplementary Figure S4b). After sequencing the transcribed loci, we mapped them to the human genome and achieved a mapping rate of more than 90% across all samples. We then called variants across loci, filtered out indels, multi-allelic variants, variants with low counts, and retained a total of 94 of the originally identified SNP-gene pairs for differential analysis. Replications for each sample were highly correlated (*r*^2^ ≥ 0.9) (Supplementary Figure S4c).

Because all samples shared the same input library, we focused on analyzing differences of STARR-seq transcriptional activities among drug treatment conditions and alleles. Principal component analysis showed global differences among drug treatments (Supplementary Figure S4d), demonstrating drug effects on STARR-seq transcription. Particularly, differential analysis showed that 95% of cloned loci exhibited drug-dependent transcription activity across cell lines and drugs (Supplementary Figure S5a–d, Table S2). To test for allele-dependent drug-responsive effects, we analyzed percentage differences of alternative alleles before and after drug treatments. In LCLs, the expression of 44 originally genotyped SNPs was allele-dependent after cortisol treatment, as were 55 after C297 treatment (FDR < 0.05, percentage change of alternative allele > 10%) (Figure 4B). *De novo* SNPs identified by sequencing in STARR-seq demonstrated that 55 (cortisol) and 67 (C297) loci had allele- and drug-dependent transcriptional activity (Figure 4C). In A549 cells, expression of 52 originally genotyped SNPs was allele-dependent after cortisol treatment, and 61 after C297 treatment (Figure 4D). *De novo* SNPs identified by sequencing in STARR-seq demonstrated that 62 (cortisol) and 73 (C297) loci had allele- and drug-dependent transcriptional activity (Figure 4E). Out of 66 allele- and drug-dependent loci where *de novo* SNPs were discovered, 54 (82%) had *de novo* SNPs that were in LD with the original genotyped SNPs based on 1000 Genome Project Phase I (see Supplementary Data 2 for more details). These loci displayed high consistency between the two cell lines in which STARR-seq was applied (Figure 4F). As anticipated, the majority of allele- and drug-dependent loci identified by STARR-seq were ChromHMM-predicted enhancers, validating up to 81% of the cloned PGx loci that we had identified (Figure 4G, see Supplementary Data 2 for details on each locus). Overall, up to 94% of PGx loci also harbored GR-dependent transcription activity, demonstrating robust functional modulation of GR in these loci (Supplementary Figure S5e). We also further validated selected STARR-seq SNP loci that later displayed associations with clinical phenotypes (see below) with traditional luciferase reporter gene assays and observed similar allele- and drug-dependent activity between the two assays (Supplementary Figure S6).

### PGx-eQTL SNP-gene pairs are connected by drug-dependent enhancer-enhancer and enhancer-promoter loops

**Figure 5.**
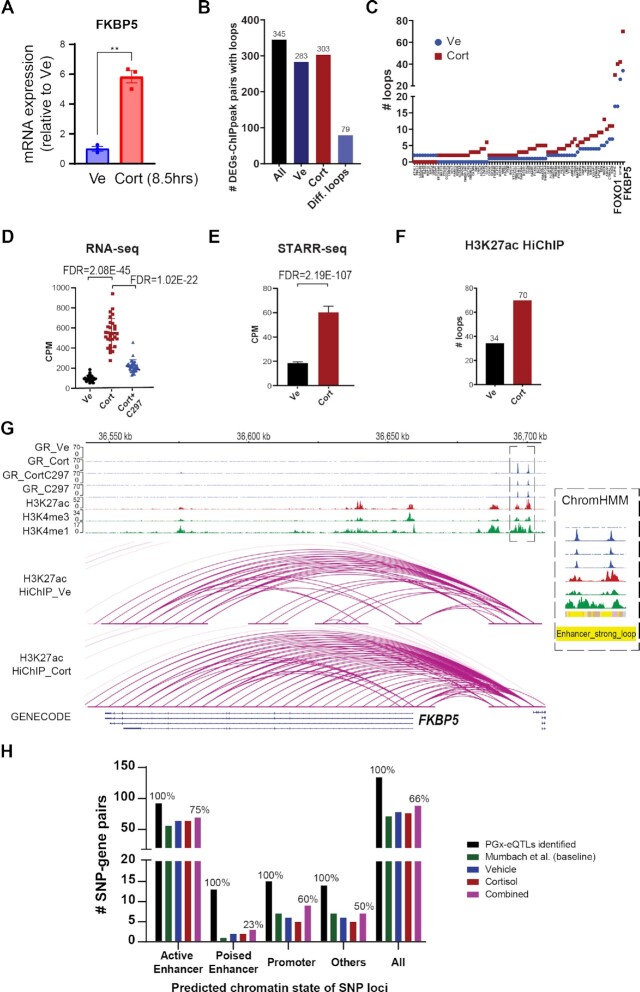
**Generation and integration of H3K27ac HiChIP before and after glucocorticoid treatment with other datasets**. (**A**) Validation of drug treatment effect for HiChIP samples as measured by qRT-PCR for *FKBP5*, a prototypical GR-targeted gene, using RNA extracted from the same cells. Statistical significance was evaluated with Student's t-test, achieving *P* < 0.005. Each dot represents a replicate. (**B**) Number of cortisol-regulated genes that were connected to a cortisol-induced GR ChIP-peak by a H3K27ac loop in different treatment conditions. (**C**) Cortisol-regulated genes that were connected to one or more cortisol-induced GR ChIP-peaks by differential cortisol-regulated H3K27ac loop(s) (fold change > 1.5 or a change from 0 that is more than 1). The X axis shows gene names (Please see Supplementary Data 1 for a complete list of gene names). (**D**) mRNA expression of *FKBP5* before and after drug treatment as determined by RNA-seq. CPM represents counts per million. (**E**) Transcriptional activity driven by the enhancer region upstream of *FKBP5* as measured by STARR-seq. (**F**) Number of HiChIP H3K27ac loops that connected GR-binding sites to the *FKBP5* gene. (**G**) Integrative Genomics Viewer (IGV) plots of two different GR-dependent enhancers over a distance of 50kb, which together regulated *FKBP5*. These two enhancers were predicted to be strong enhancers with looping properties by ChromHMM. (**H**) Number of PGx-eQTLs that displayed physical interactions between SNP loci (categorized by enhancer/non-enhancer states) and eQTL genes as demonstrated by H3K27ac HiChIP.

To determine the nature of physical interactions between PGx SNP loci and eQTL genes, we applied H3K27ac HiChIP, an assay that can capture chromatin conformation of enhancer-promoter and enhancer-enhancer interactions in a high-resolution manner (29), before and after cortisol exposure. First, to demonstrate that drug treatment in the HiChIP experiment was successful, qRT-PCR was conducted for *FKBP5*, a prototypical GR-targeted gene, using total RNA from the same cells before fixation. *FKBP5* was induced ∼6-fold after drug treatment, confirming drug treatment effect (Figure 5A). After library preparation, H3K27ac ChIP efficiency was achieved at 0.21% to 0.42% of total input for vehicle and cortisol, respectively. Shallow sequencing confirmed that the percentage of PCR duplication was less than 0.01%, and that more than 40% of the fraction of reads represented interactions within the same chromosome (long-range *cis* interactions). Deep sequencing yielded 829,599,511 raw PE reads for vehicle (of which 88.1% were mapped), and 539 417 612 for cortisol (of which 87.4% were mapped). Using known ENCODE H3K27ac ChIP-peaks for LCLs for loop calling at an FDR threshold of 0.01, the number of called loops was 193,107 for vehicle and 131,926 for cortisol, which displayed high enrichment around ChIP-peaks (Supplementary Figure S7a). These numbers are comparable with those reported for a publicly available H3K27ac HiChIP library for LCL GM12878 (185,167 loops) (29). The percentage of intra-chromosomal interactions that spanned >15 kb in linear genomic distance, a measure of how well the library captured chromatin interactions between genomic loci, was 35.7% for vehicle and 36.9% for cortisol, surpassing the manufacturer's minimum benchmark of 25%. Percentages of valid interaction pairs located within known ChIP-seq peaks was 37.5% for vehicle and 33.5% for cortisol, surpassing the manufacturer's minimum benchmark of 15%.

To determine whether H3K27ac loops changed after cortisol treatment and to what extent they might be correlated with functional outcome (differential gene expression), we integrated the cortisol-regulated RNA-seq, GR ChIP-seq and HiChIP datasets. Out of 1361 differentially expressed genes (DEGs), 345 had HiChIP loops connecting them to one or more cortisol-induced ChIP-seq peaks, and 79 DEGs had loops that were altered after drug treatment (defined as a fold change in number of loops >1.5 or a change from no loop to a number > 1) (Figure 5B, C). To help readers visualize this integrative approach, which was applied later to fine-map PGx-eQTL SNP-gene pairs, we showcased two positive controls for all of the datasets in Figure 5D-G and Supplementary Figure S7b-d. Specifically, RNA-seq showed that *FKBP5* mRNA expression was induced by cortisol (FDR *=*2.08E−45), an induction that was reversed by C297 (FDR *=*1.02E−22). This observation for gene expression correlated with the GR-binding patterns under the same drug conditions at the enhancer regions that mapped 50kb upstream of *FKBP5* (Figure 5D, E). This region was predicted to be a strong enhancer with looping properties (Figure 5G). After being tested in STARR-seq, it showed a strong induction in enhancer activity after drug treatment (FDR = 2.19E−107) (Figure 5E). The number of H3K27ac HiChIP loops also increased 2-fold after cortisol treatment, connecting the GR-induced enhancers to *FKBP5*, transcriptionally regulating the gene (Figure 5F). Similar observations were made for *FOXO1*, for which GR-modulated enhancer signals were integrated from four different regions over hundreds of kilobases, with a 2-fold change of HiChIP loops after drug treatment (Supplementary Figure S7b–d).

As expected, most of the SNPs that were connected with PGx-eQTL genes with H3K27ac loops mapped within ChromHMM-predicted active enhancers (Figure 5H). Publicly available data (29) showed that 71 of the PGx-eQTLs that we identified displayed H3K27ac connecting ‘loops’ at baseline. We observed a similar number of connected PGx-eQTLs before and after cortisol treatments in our data (78 and 76, respectively) (Figure 5H), with 68 being constitutive loops connecting the PGx SNP loci and eQTL genes but requiring glucocorticoids to ‘unmask’ their functional transcriptional regulation. In fewer cases, cortisol was shown to induce loops for 5 SNP-gene pairs or to repress loops for 6, either bringing together SNP-gene loci that otherwise would not have been in proximity or separating them (Supplementary Data 2). The precise molecular mechanisms by which GR reorganizes the enhancer landscape have been described elsewhere (46).

### Glucocorticoid-modulated PGx-eQTLs unmasked potential function of SNP loci previously associated with common diseases involving glucocorticoid signaling

**Figure 6.**
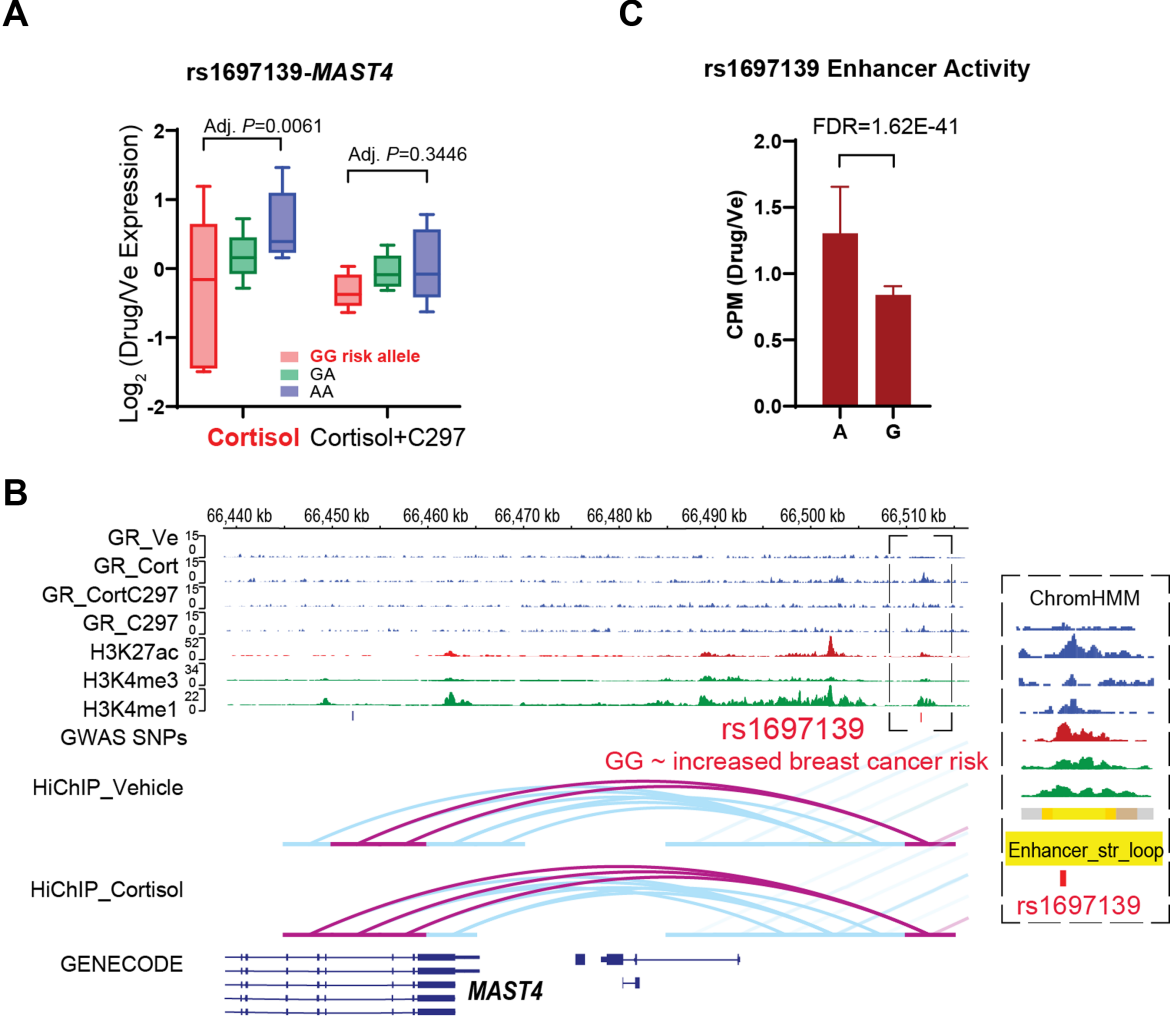
**A** **n example of GR-dependent PGx-eQTLs with functional implications for disease risk**. (**A**) PGx-eQTL SNP-gene pairs for rs1697139-*MAST4*. Adjusted *P*-values from Tukey's *post-hoc* multiple comparisons and represent differences between wildtype and variant genotypes. Cortisol induced the eQTL, and C297 antagonized the cortisol effect, normalizing eQTL expression across genotypes. (**B**) IGV plots of the PGx SNP-eQTL gene locus. Tracks for GR-targeted ChIP-seq in different treatment conditions are colored in blue, which show similar drug-dependent patterns as expression data: Cortisol induced GR binding at SNP locus, and C297 antagonized the cortisol effect, reducing GR binding. H3K4me1 is a histone mark associated with enhancers. H3K4me3 is a histone mark associated with promoters. H3K27ac is a histone mark associated with active promoters and enhancers. For the H3K27ac HiChIP tracks, loops directly interacting with the PGx SNP locus are highlighted in pink and others in blue. (**C**) SNP-dependent and drug-dependent enhancer activity of the PGx locus in LCLs as measured by STARR-seq. CPM stands for counts per million.

**Figure 7.**
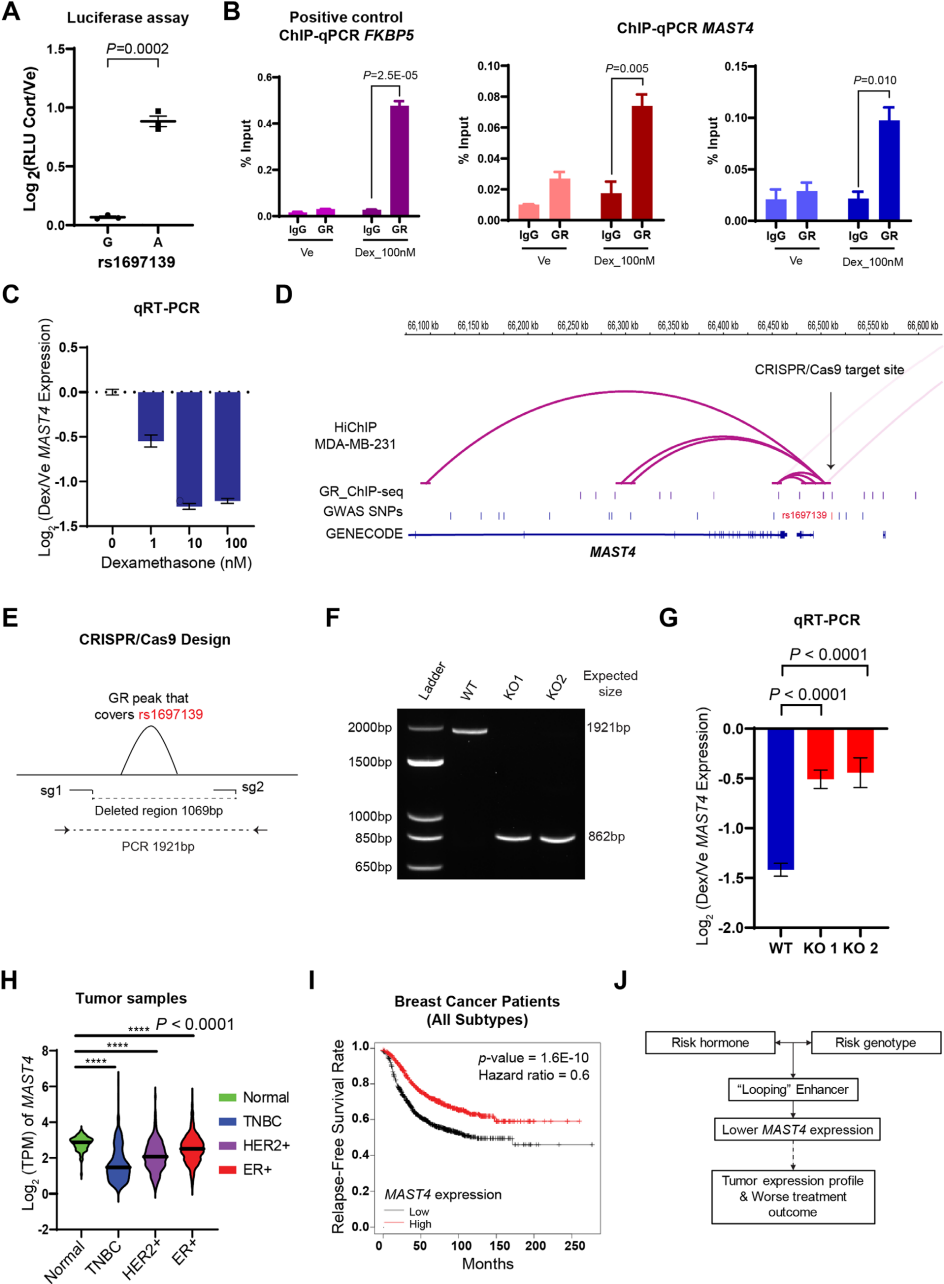
**Functional Validation of rs1697139-*MAST4* GR-dependent PGx-eQTL in Breast Cancer Cells**. (**A**) SNP-dependent and drug-dependent enhancer activity of the PGx locus associated with breast cancer in Figure 6 as measured by luciferase reporter gene assay in MDA-MB-231, a triple negative breast cancer cell line. RLU stands for relative light units and reflects normalization of luciferase signal to Renilla signal as an internal control. (**B**) ChIP-qPCR assays that tested GR binding at the PGx locus for MAST4 in MDA-MB-231 using two different primers. ChIP-qPCR targeting *FKBP5* peak served as a positive control. *P*-values from Student's t-tests. (**C**) Dose-dependent repression of *MAST4* by glucocorticoids in MDA-MB-231 cells. (**D**) Epigenomic datasets for MDA-MB-231 cells demonstrated that the SNP locus also looped to *MAST4* in MDA-MB-231. GR ChIP-seq was downloaded from the database ReMap2022. (**E**) Designs of guide RNAs utilized in the CRISPR/Cas9 experiment that cut out the PGx SNP enhancer locus where GR binds and primers to test editing outcomes. (**F**) Gel electrophoresis of PCR products amplified with primers targeting the cut region demonstrated successful edits. (**G**) *MAST4* gene expression as measured by qRT-PCR after CRISPR/Cas9 edits showed that the glucocorticoid-dependent repression was alleviated after the PGx SNP locus was cut out across two colonies of pure knock-out cells. Data included three biological replicates. (**H**) Expression of *MAST4* in tumors from breast cancer patients in the TCGA database. **** *P*-values < 0.0001 by Student's *t*-tests. TPM stands for transcripts per million. (**I**) Kaplan–Meier curves for relapse-free survival rate in 1764 breast cancer patients predicted based on *MAST4* expression. (**J**) Summary of functional insights gaining from in-depth studies of a GR-dependent eQTL and their implications for breast cancer risk.

In an unbiased search of GWAS/PheWAS databases (https://www.ebi.ac.uk/gwas/, https://pheweb.sph.umich.edu/, https://r4.finngen.fi/about), we found that twenty-five percent of the glucocorticoid-modulated PGx-eQTL SNPs that we identified had previously been associated with clinical phenotypes, but usually without a clear underlying mechanism and often lacking clarity with regard to the gene or genes involved. These associations spanned many different disease and drug response categories including adverse response to corticosteroids, inflammation and immunity, osteoporosis, neuropsychiatric disorders and cancer (Table 1; see Supplementary Data 2 for integrative annotation of each locus). For example, a mechanistically unexplained variant that had been associated with breast cancer risk, rs1697139 (26), was a cortisol-dependent PGx-eQTL for the Microtubule Associated Serine/Threonine Kinase Family Member 4 (*MAST4*) gene. Specifically, *MAST4* expression was repressed by cortisol in subjects with the G/G but not the A/A genotype (Adjusted *P* = 0.0061), and that repression was reversed after antagonist treatment (adjusted *P* = 0.3446) (Figure 6A). This SNP, in a genotype-dependent fashion, modulated a GR-responsive intergenic enhancer (FDR = 1.62E−41) that ‘looped’ across 40,000 bp to *MAST4*, transcriptionally regulating that gene (Figure 6B, C). Because these sets of data were generated from LCLs, we conducted a series of functional studies to validate the findings in cell lines that were directly relevant to the disease. We found that rs1697139 interfered with GR-dependent enhancer activity in MDA-MB-231 cells, a triple-negative breast cancer cell line, in a way similar to that observed in LCLs (Figure 7A). We also demonstrated that GR bound to the same rs1697139 locus and dramatically repressed *MAST4* expression in MDA-MB-231 cells as well as cell lines for other breast cancer subtypes (Figure 7B, C, Supplementary Figure S8a−f). Additionally, the SNP locus also appeared to loop to *MAST4* in MDA-MB-231 based on HiChIP data (Figure 7D). To interrogate the causal relationship between this SNP locus and *MAST4* gene expression in breast cancer cells, we used CRISPR/Cas9 to delete the SNP region (Figure 7E, F) and measured GR-dependent *MAST4* mRNA expression. We observed a decrease in the repression of *MAST4* by GR across different colonies of pure knock-out cells (Figure 7G). Although the function of *MAST4* in breast cancer is unknown, it may be a novel risk gene since its expression was significantly repressed in breast tumor tissue when compared with normal breast tissue (*P* < 0.0001) (Figure 7H). Furthermore, *MAST4* expression also appeared to be a predictor of treatment response since decreased expression of *MAST4* was associated with decreased relapse-free survival (*P* = 1.6E−10) (Figure 7I). These observations agree with the direction of the SNP-phenotype association. Specifically, the rs1697139-G/G genotype was associated with decreased *MAST4* expression after exposure to cortisol, a hormone that promotes breast cancer heterogeneity and metastasis (16), which might have led to the increased risk for breast cancer observed in the original GWAS (Figure 7J). Of interest is the fact that glucocorticoids were recently found to induce chemo-resistance in solid tumors by transcriptionally regulating another MAST family member, *MAST1* (47), a protein which has the most homology with *MAST4* within this protein family (48).

Beyond this illustrative example, Table 1 lists a total of 30 disease risk loci that we found to behave as PGx-eQTLs (see Supplementary Table S4 for current knowledge of each gene). Of importance, glucocorticoids were either known risk factors or therapeutic agents used to treat most of these diseases, demonstrating a genetic risk × hormone/drug risk interaction in disease predisposition. For instance, glucocorticoids are potent immune-suppression and anti-inflammatory agents (13). As a result, GR agonists are used clinically to treat a wide variety of immune-related diseases including those listed in Table 1—e.g. multiple sclerosis (18) and systemic lupus erythematosus (19). Corticosteroids are also known to increase platelet number and, as a result, are first-line therapy for immune thrombocytopenia (49). In another example, we found that a SNP previously associated with response to vaccine and counts for different immune-related blood-cell types interacted with cortisol to influence the expression of RUNX Family Transcription Factor 1, *RUNX1* (Supplementary Figure S9a), a major regulator of hematopoiesis (50). Specifically, the *RUNX1* G/G genotype increased GR binding, correlating with an increase in GR-modulated enhancer activity, an intergenic enhancer that ‘looped’ the *RUNX1* promoter, resulting in decreased *RUNX1* expression (Supplementary Figure S9b–e). The repression of *RUNX1* could result in a decrease of B cells (50), which might help to explain, in part, why antibody levels after vaccination were decreased in individuals carrying the G/G genotype (51). Different from the breast cancer example presented above, where the direction of the genotypes were the same between enhancer activity and gene expression (lower activity, lower expression), in this example for *RUNX1*, we observed that the risk allele was associated with higher STARR-seq cortisol induction but reduced *RUNX1* expression. Because results from luciferase reporter gene assay independently validated the direction of the SNPs, this ‘discrepancy’ was not due to technical error of the assay but perhaps some other type of biological mechanism, such as (i) the indirect effect of the SNP on *RUNX1* via other nearby interacting genes such as *CLIC6* (Supplementary Figure S9e), and/or (ii) the effect of multiple regulatory elements in the chromatin context that reporter assays are unable to capture (26). We also identified a SNP previously associated with lichen planus, an autoimmune condition that attacks cells of the skin and mucous membranes, that interacted with cortisol to influence the expression of *NLRC5*, a key regulator of adaptive immune responses (52,53).

**Table 1.** GR-modulated PGx-eQTLs which we identified that have been associated with clinical phenotypes by previous GWAS/PheWAS. SNP-gene pairs that were PGx-eQTLs (*P* < 0.05) but not eQTLs in GTEx (*P* > 0.05) are bolded

Genotyped SNP	Ligands	PGx-eQTL gene(s) Identified	SNP-associated phenotype(s) in GWAS/PheWAS
**Pharmacogenomics**			
rs11633087/ rs4843073/ rs4843074/ rs4843075/ rs7162168/	Cortisol	*KLHL25*	Adrenal cortical steroids causing adverse effects in therapeutic use (61)
**Inflammation/Immunity**			
rs2834655	Cortisol	*RUNX1*	Immune response to smallpox vaccine (51) Monocyte percentage of white cells (62) Red blood cell count (63)
**rs2984920/** **rs7535818**	Cortisol	***RGS1***	Systemic lupus erythematosus risk (64) Intestinal malabsorption/Celilac disease (61) Multiple sclerosis (65) Dermatitis and eczema*
rs4735336	Cortisol	*PLEKHF2*	Viral hepatitis C (61)
**rs2297539**	Cortisol	*IKBKE/* ***SRGAP2***	Fasciitis Celilac disease (61)
**rs2399594**	Cortisol	***NLRC5***	Lichen Planus (inflammation of the skin)*
**rs12440899**	Cortisol	***SRP14-AS1***	Inflammatory liver diseases*
**rs12053126**	CORT108297	***AFF3***	Otosclerosis*
**rs9594738**	CORT108297	***DGKH***	Otosclerosis*
**rs2051541**	Cortisol	***HIST1H2AC***	Celilac disease (61) Ankylosing spondylitis (inflammation of the spine)* Acute and subacute iridocyclitis (inflammation of the iris)*
**rs7356**	Cortisol	***EYA3***	Platelet count (66)
rs4843073	Cortisol	*KLHL25*	Neutrophil count (62)
**rs4984913**	CORT108297	***CCDC78***/***WDR90***/***FAM195A***/ ***WFIKKN1***	Platelet count (62)
**Psychiatry & Neurology**			
**rs2779180**	Cortisol	***ARRDC5***	Depression in response to interferon-based therapy of chronic hepatitis C (67)
**rs1050863**	Cortisol	***ZCCHC14*** */* ***JPH3***	Mood Instability (68)
**rs696284**	CORT108297	***NFX1***	Mood disorders*
**rs7288411**	Cortisol	***RRP7BP***	Bipolar disorder or major depressive disorder (69)
**rs11678116**	Cortisol	***LDAH***	Migraine*
**Cancer**			
**rs1697139**	Cortisol	***MAST4***	Breast cancer risk(26)
**Bone-related diseases**			
**rs9594738**	CORT108297	***DGKH***	Bone mineral density (70) Medication use (drugs affecting bone structure and mineralization) (71) Otosclerosis*
**rs12053126**	Cortisol	***AFF3***	Otosclerosis*
**rs1539330**	Cortisol	***KIF11***	Fracture of hand or wrist (61)
**rs7406439**	Cortisol	***HEXDC***	Disorders of continuity of bone*
**Metabolism**			
**rs1978487**	Cortisol	***FBXL19/HSD3B7***	Obesity/hypertension/Body mass index (61)
**rs11215427**	Cortisol	***CADM1***	Childhood body mass index (72)
rs4735336/ rs4735337	Cortisol	*PLEKHF2*	Hypertensive diseases*
**Others**			
**rs2051541**	Cortisol	***HIST1H2AC***	Disorder of iron metabolism (61) Hereditary hemochromatosis (61)
**rs10053292**	Cortisol	***SLC26A2***	Acquired deformities of finger (2)
**rs12440899**	Cortisol	***SRP14-AS1***	Endometriosis*
rs2313167	CORT108297	*PDLIM2*	Height (2)

*Associations from FinnGen PheWAS.

In addition to inflammation and immunity, glucocorticoids also play an important role in osteoporosis (14), mood disorders (8,15), cancer (16) (as described above), and metabolism (17), diseases that are also listed in Table 1. In an example involving the rs11678116 SNP and the Lipid-Droplet Associated Hydrolase (*LDAH*) gene, cortisol was shown to bring together SNP-gene loci that otherwise would not have been in proximity, ‘unmasking’ the impact of rs11678116 on *LDAH* transcription (Supplementary Figure S10a, b). Specifically, the rs11678116 SNP created a GR binding motif (Supplementary Data 2), which led to decreased cortisol-responsive enhancer activity of the T/T genotype and decreased *LDAH* expression after cortisol treatment of subjects with the T/T but not the G/G genotype (Supplementary Figure S10c). Based on PheWAS results, rs11678116 was also associated with migraine (Table 1), a stress-sensitive condition for which cortisol is a biomarker (54), providing an intriguing genotype-phenotype link for functional investigation given the elevated levels of cholesterol and triglycerides observed in migraine patients (55). We also found that SNPs previously associated with body mass index and obesity interacted with cortisol to influence genes located as far as 150,000bp away such as *FBXL19*, an adipogenesis-controlling gene (56), *HSD3B7*, a cholesterol metabolizing enzyme (Supplementary Figure S11a, b), or *CADM1*, a gene that regulates body weight via neuronal modulation (57). Taken together, these examples demonstrate that glucocorticoid-dependent PGx-eQTLs identified in LCLs uncovered functional SNPs related not only to immune-related diseases but also diseases reflecting dysfunction of various other cell types. That is consistent with previous observations that eQTLs which are shared across tissues comprise a larger fraction of trait associations than do tissue-specific eQTLs (58).

## DISCUSSION

Despite the many associations with human disease that have been described for non-coding genetic variants, their functional interpretation remains a significant challenge. Furthermore, complex diseases are usually influenced by both genetic and environmental factors, which are difficult to interrogate mechanistically (11). This manuscript addresses these two themes by mechanistic studies of a type of non-coding genetic variant with functions that are modulated by pharmacologic or physiologic chemical agents. Obviously, this study has limitations previously discussed that are inherent to the study of response-eQTLs (4)—namely, limited cell types that are available as study models and limited power because of the resources required to generate datasets with and without chemical stimuli. We addressed these issues by studying combinations of treatment with agonists and antagonists to verify drug-dependent effects. We then provided additional layers of mechanistic evidence to support our observations but with the clear acknowledgement that we might fail to capture all relevant SNPs. We also validated selected examples with experiments in other relevant cell lines, coupled with the integration of available clinical data for functional interpretation. For example, we conducted a series of in-depth functional validation studies using breast cancer cell lines for a breast-cancer-risk SNP that was discovered in LCLs to be a PGx-eQTL with *MAST4* (Figures 6 and 7). We found that the SNP behaved in a similar fashion in MDA-MB-231, a triple negative breast cancer cell line, in terms of its interaction with glucocorticoids and the subsequent impact on *MAST4* expression. Furthermore, the more prominent repression of *MAST4* in cell lines carrying the risk genotype by glucocorticoids, a risk hormone for breast cancer metastasis (16), fits with the fact that lower expression of *MAST4* was observed in breast cancer tissues and was associated with worse treatment outcomes in breast cancer patients. This example demonstrated that, while glucocorticoid-dependent PGx-eQTLs were identified in LCLs, novel functional genes could be uncovered for not only immune-related diseases but also diseases reflecting dysfunction of other cell types, encouraging similar functional validation of clinically significant signals in relevant cell types and datasets in the future and the development of new peripheral biomarkers for diseases and therapeutic responses. Moreover, functional studies of genes discovered via this mechanism have already yielded novel insights into mechanisms of disease (6,8), serving as a logical next step after novel risk genes have been identified during SNP-focused studies. The mechanistic insight that certain SNPs only manifest their function after exposure to particular chemical stimuli might also encourage a modified approach in GWAS studies in which these environmental factors are built into SNP-phenotype association models.

We observed that the function of these PGx-eQTL SNPs was usually ‘masked’ in the absence of exposure to hormones or related drugs because exposure to these compounds could either initiate transcriptional activity between connected loci or elicit a conformational change in the epigenomic landscape to disrupt or bring the SNP locus into contact with distal gene(s) that were often relevant to the observed clinical phenotypes. While we observed that GR could mediate chromatin looping dynamics similarly to what has been described by Hoffman *et al.* (46), most SNP-gene pairs appeared to have pre-established connections at baseline, an observation consistent with that reported by D’Ippolito *et al.* (59). A limitation of Hi-C-based techniques, however, is that it provides a static snapshot of chromatin interactions, interactions which have been shown to be overwhelmingly transient and dynamic by super-resolution live-cell imaging (60).

Another relevant question to be explored is whether these GR-responsive eQTLs could be replicated *in vivo* in human studies. To partially address this issue, we overlapped our PGx-eQTL discoveries with the 296 unique SNP-gene pairs described by Arloth *et al.* (10) that were identified from peripheral blood mononuclear cells (PBMCs) of psychiatric patients after dexamethasone stimulation. However, given the large differences in experimental designs between the two studies (e.g. microarray versus RNA-seq, PBMCs versus B-lymphocytes, varying blood drug levels in patients versus tightly controlled drug conditions in cell lines), we were unable to observe an overlap.

In conclusion, by systematically fine-mapping genotype-phenotype interactions in which measurable environmental factors such as drug or hormone exposure were taken into account, we uncovered potential novel risk genes for a wide range of diseases in which the pharmacological or physiological stimuli played important roles. As a result, this study has added a novel perspective to functional genomics by providing a mechanistic framework for additional studies of ligand-dependent ‘silent’ non-coding genetic variants to advance the fine-mapping of disease risk and pharmacogenomic loci. Insights from those efforts could partly explain mechanisms underlying genetic and environmental susceptibility to common diseases and/or predict variation in response to drug therapy.

## DATA AVAILABILITY

All sequencing data was deposited on Gene Expression Omnibus under accession number GSE185941.

## Supplementary Material

gkac1045_Supplemental_Files
