# Supplementary material for: Glucocorticoids unmask silent non-coding genetic risk variants for common diseases
Source: Nucleic Acids Res. 2022 Nov 18;50(20):11635–53. doi: 10.1093/nar/gkac1045 (PMC9723631; doi:10.1093/nar/gkac1045)
Supplement: gkac1045_Supplemental_Files [file gkac1045_supplemental_files.zip › supplementary_text_accepted_revisions.docx]

**Supplementary Figures**


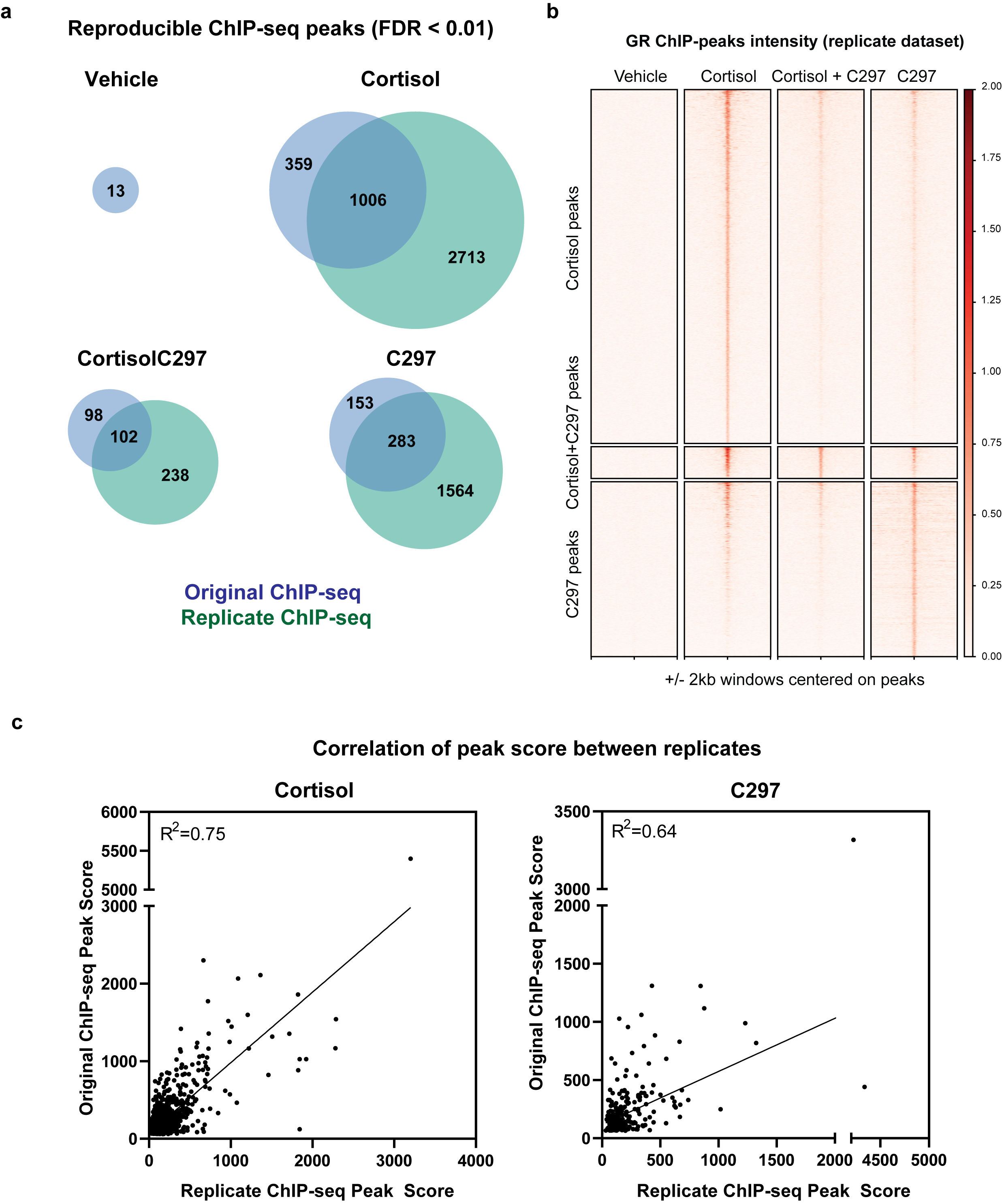


Supplementary Fig.1. Replicate of GR ChIP-seq with different antibody. (a) Overlap of peaks between the original GR-ChIPseq dataset and the replicate dataset in four drug conditions. (b) GR-targeted ChIP peak intensity after normalization to input across the 4 drug conditions shows drug-dependent patterns similar to those in the original dataset in Figure 1c. (c) Correlation of peak score for the overlapped peaks between the two datasets.


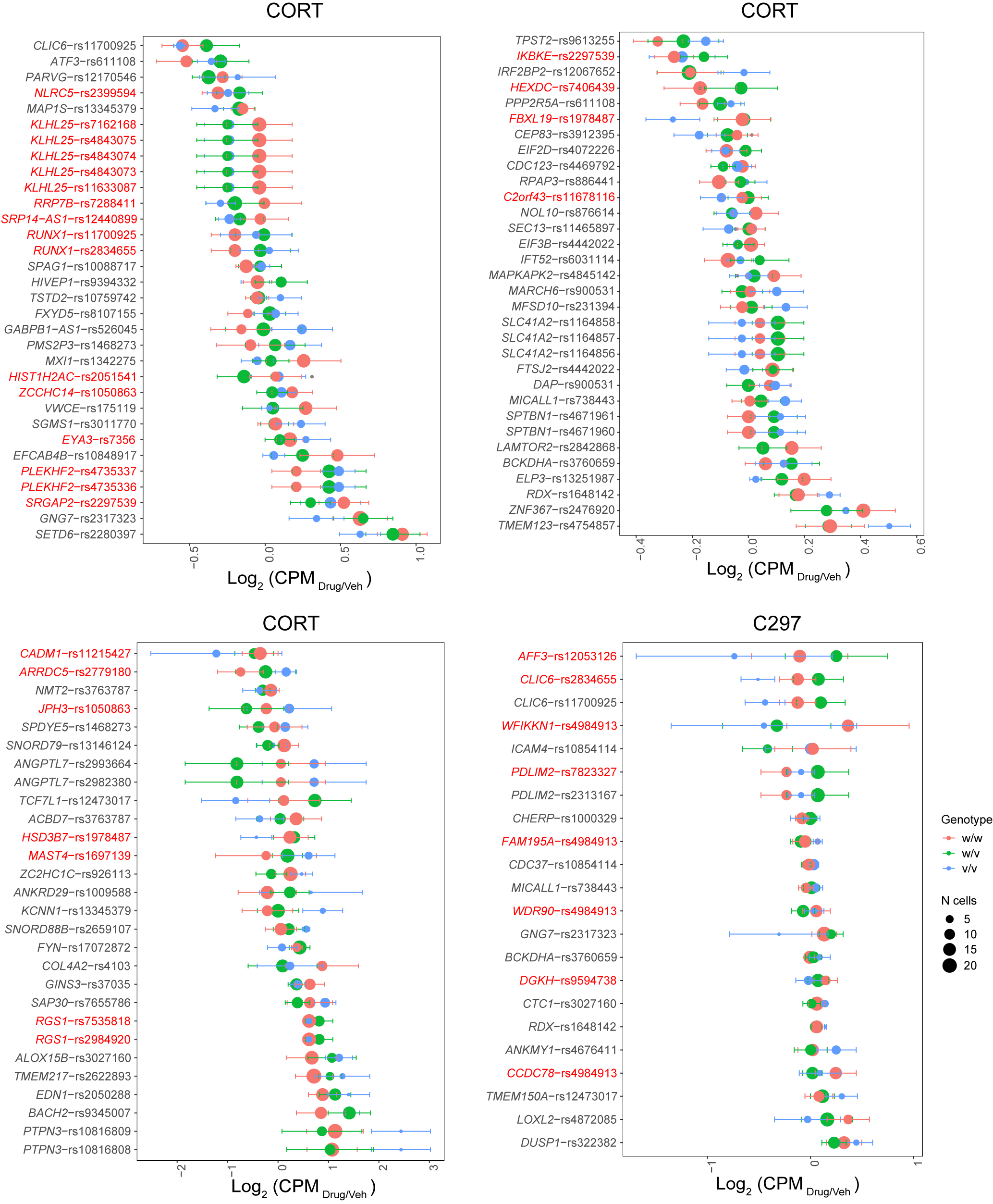


Supplementary Fig.2. Identification of GR-dependent PGx-eQTLs. Dot plots depicting eQTL gene expression patterns for drug condition normalized to vehicle. Genotypes are color coded. Dot sizes represent the number of cell lines available for each genotype. PGx-eQTL SNPs that have previously been associated with a clinical phenotype are highlighted in red text.


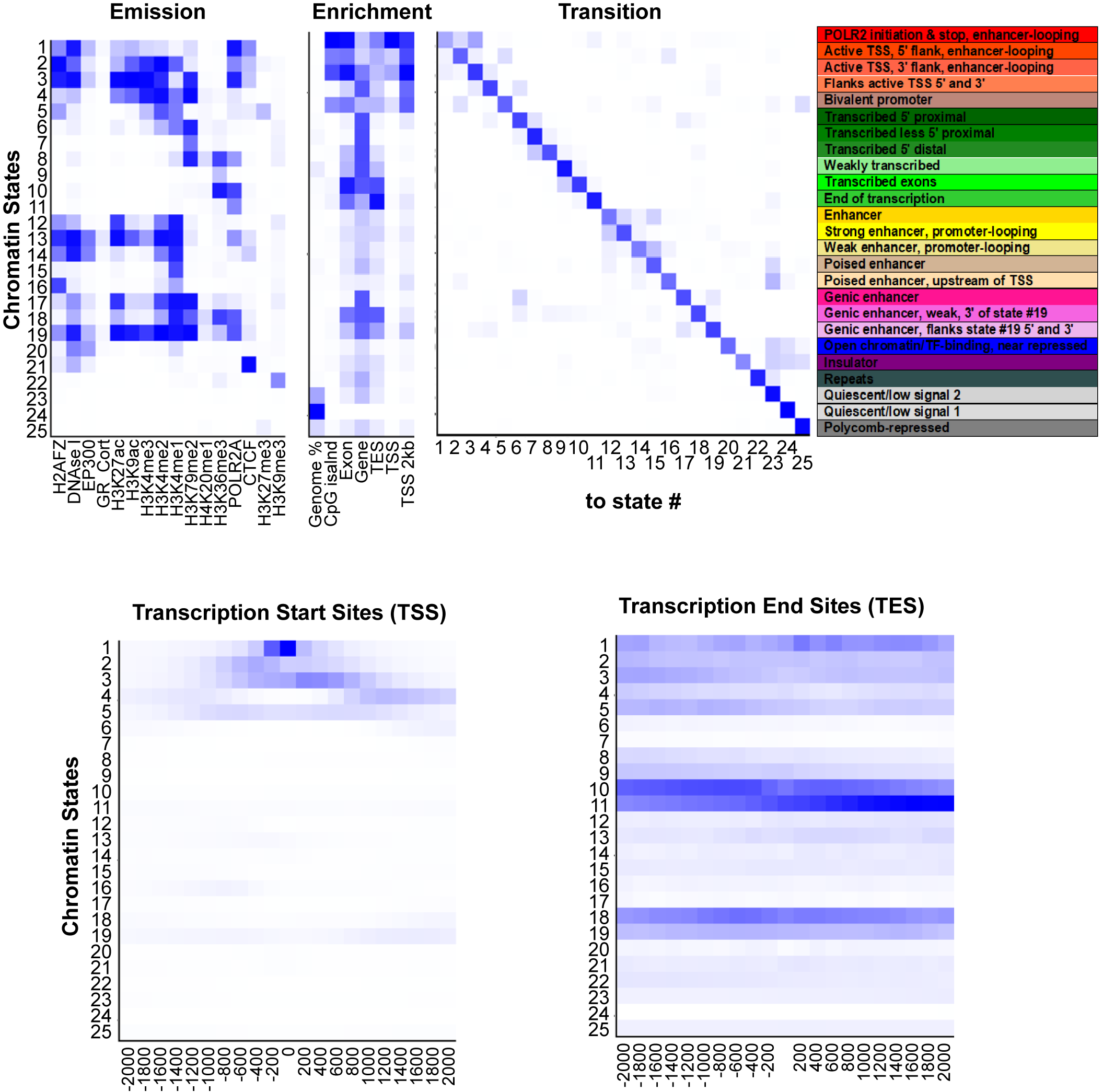
Supplementary Fig.3. Output of ChromHMM analysis. The emission panel depicts the occurrence probability for each epigenetic mark in a particular state. The combinatorial occurrence of the marks is one of the pieces of information essential for chromatin state annotation. The enrichment panel represents enrichment for each state within a particular region in the genome, with a zoom-in focus on transcription start sites and transcription end sites as shown in the lower panels. The transition panel represents the spatial proximity of each state to each other.


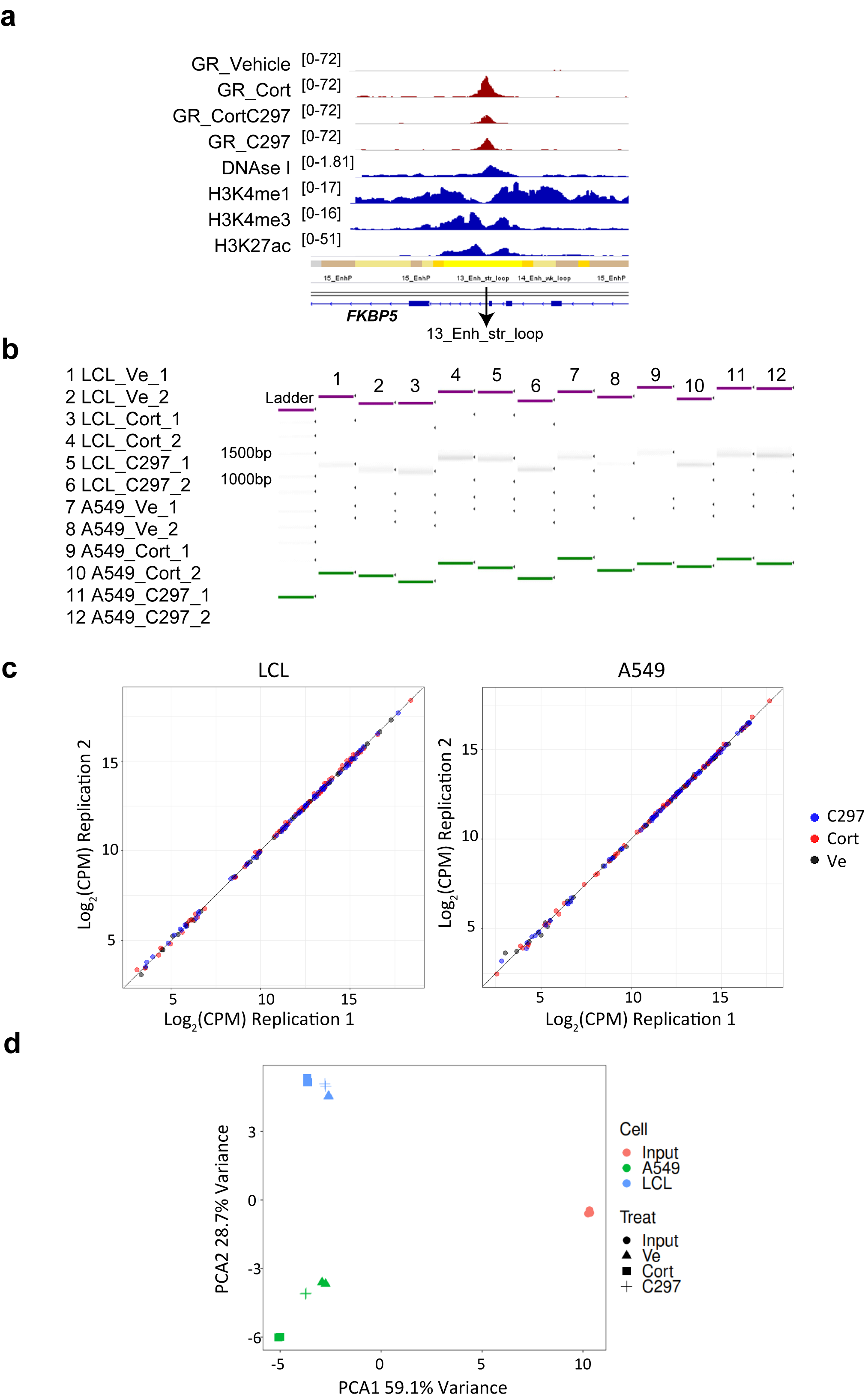


Supplementary Fig.4. STARR-seq experimental design and statistical summary. (a) A glucocorticoid-induced GR peak that mapped to a strong enhancer region with looping properties at the 5’ end of *FKBP5* was included in the STARR-seq library as a positive control for enhancer activity. (b) After RNA transcribed from STARR-seq was enriched and converted to cDNA, a PCR reaction was conducted to transform cDNA to double-stranded DNA for sequencing library preparation. The image in (b) shows the STARR-seq DNA products measured by Agilent High Sensitivity D5000 ScreenTape, which appeared around 1300bp, the expected average size. (c) Correlation of STARR-seq read counts between 2 replicates showed good replication. (d) Principal component analysis of STARR-seq results showed global differences across treatments as well as between different cell lines. Cell types are color coded. Different shapes represent different treatments.


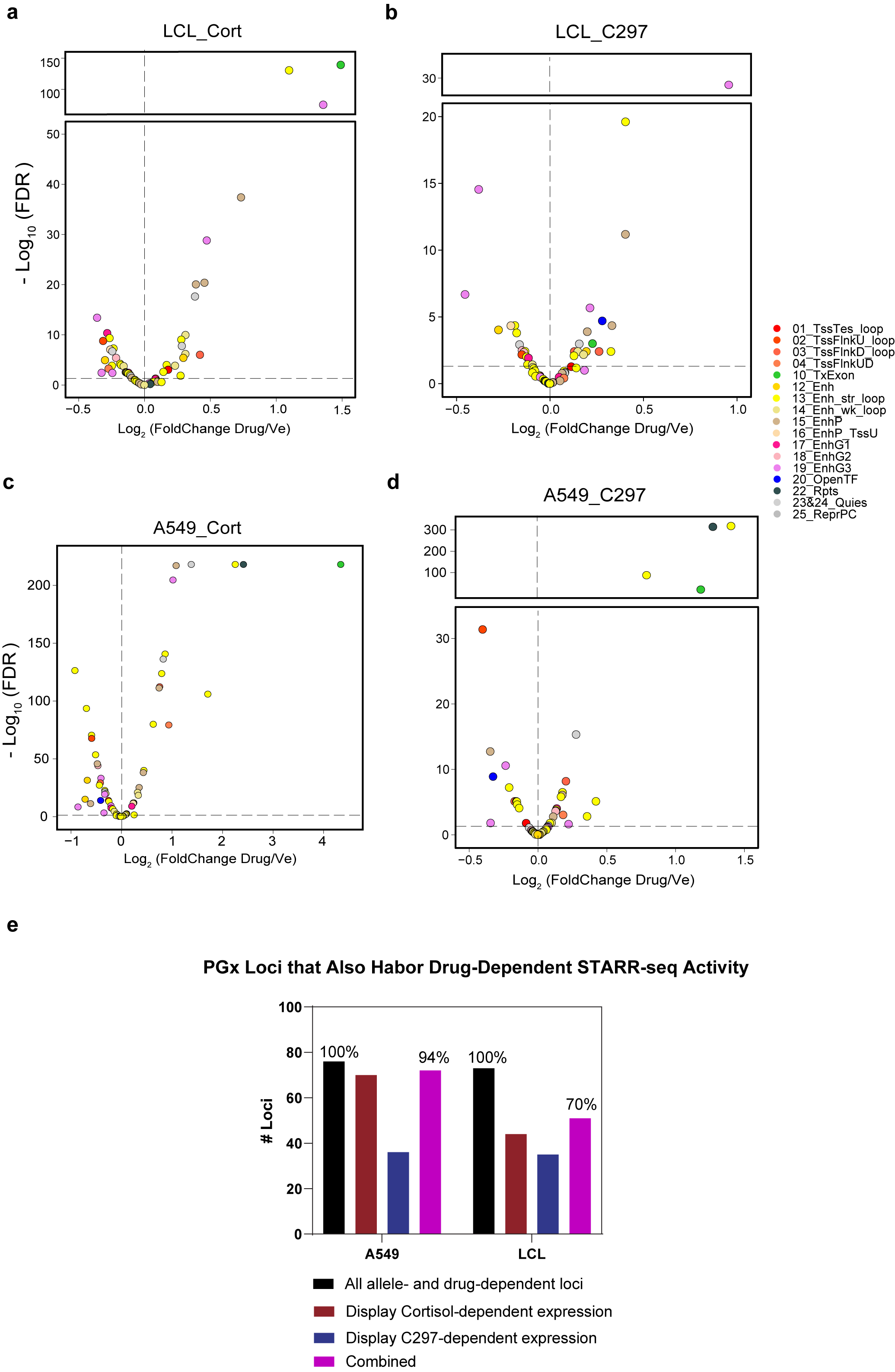


Supplementary Fig.5. Drug-dependent Properties of Cloned Loci in STARR-seq. (a-d) Volcano plots showing GR-dependent transcriptional activities of cloned loci in STARR-seq assay. X-axis represents log_2_ fold change of expression after drug treatment. Y-axis represents -log_10_ of FDR. (e) The majority of allele- and drug-dependent loci validated in STARR-seq also displayed drug-dependent activity regardless of SNP effect.


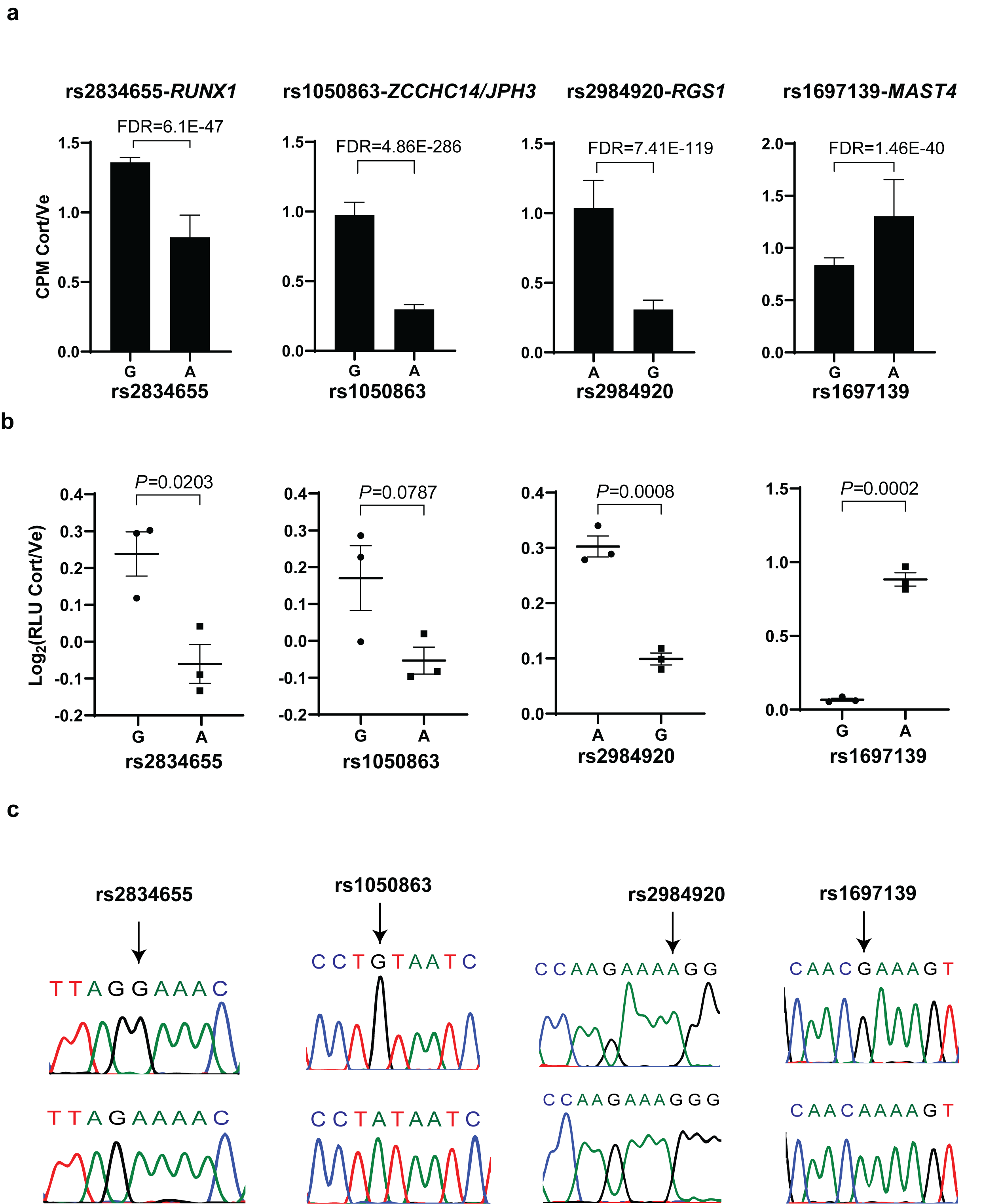


Supplementary Fig.6. STARR-seq validation by luciferase reporter gene assay for selected loci. (a) Results for each SNP locus as measured by STARR-seq. The Y-axis represents RNA-seq counts per million of cortisol normalized to vehicle. (b) Results for each SNP locus as measured by luciferase assay. The Y-axis represents luciferase/renilla ratio of cortisol normalized to vehicle. (c) Genotypes of SNPs in luciferase constructs as confirmed by Sanger sequencing.


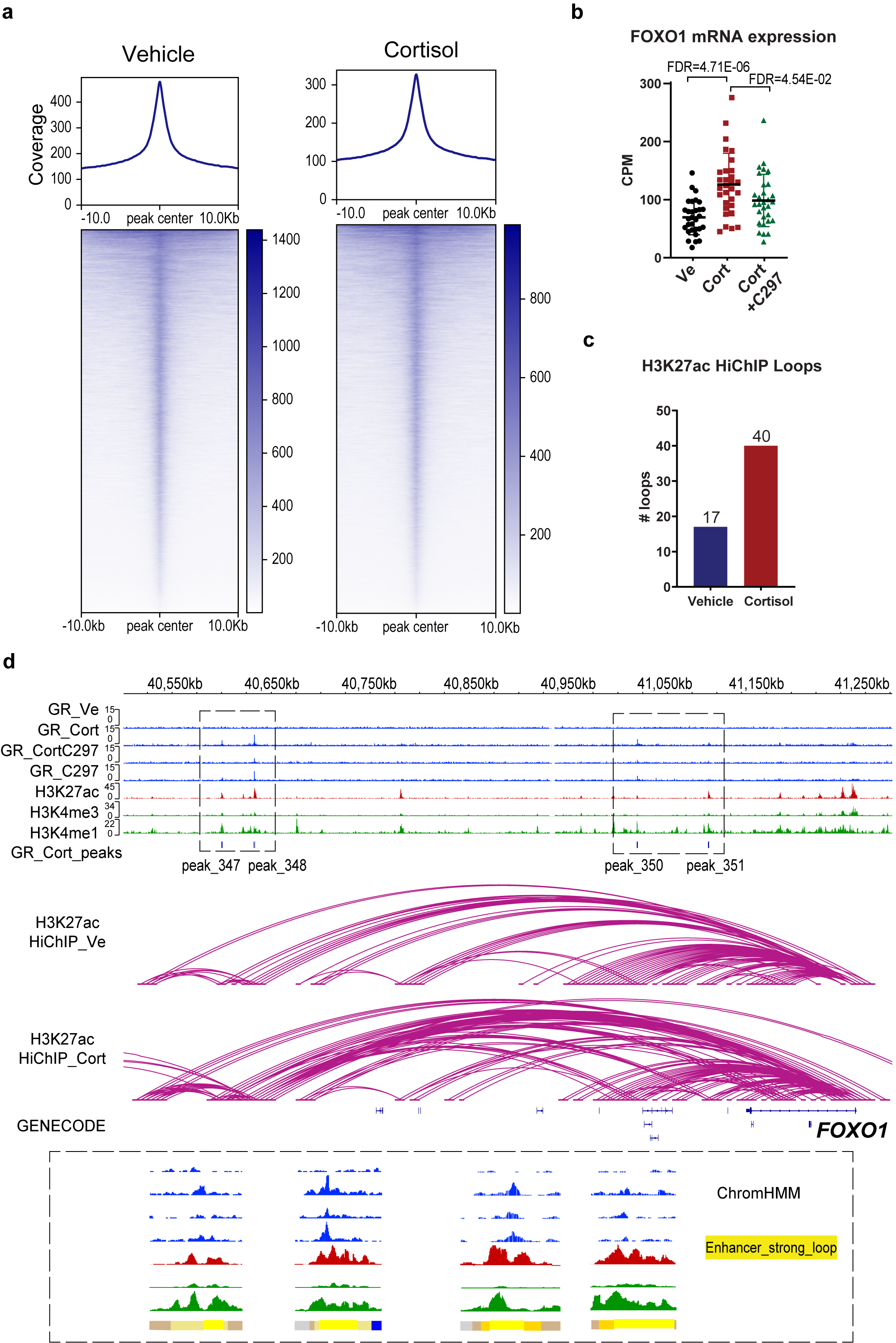


Supplementary Fig.7. HiChIP experimental controls. (a) Enrichment of loops near known H3K27ac ChIP-peaks in the GM12878 LCL from ENCODE. (b-d) Additional positive control for “-omic” datasets after drug treatment showed their integration for another prototypical GR-targeted gene *FOXO1*. (b) mRNA expression of *FOXO1* before and after drug treatment as measured by RNA-seq. CPM stands for counts per million. (c) Number of HiChIP H3K27ac loops connecting GR-binding sites to *FOXO1* gene. (d) IGV plots of 4 different GR-dependent enhancers over a distance of 600kb, which together regulated *FOXO1*. These four enhancers were all predicted to be strong enhancers with looping properties by ChromHMM.


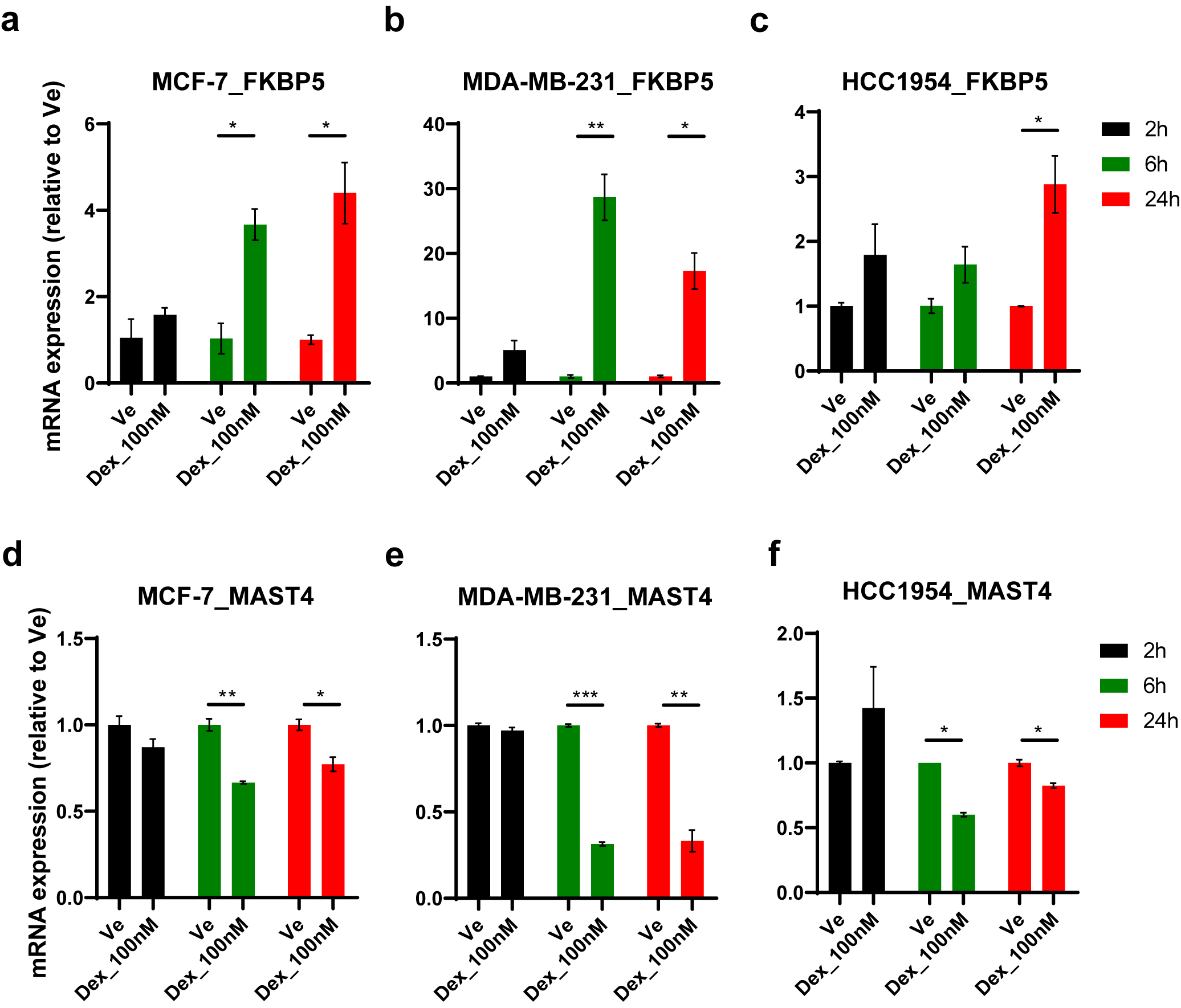


Supplementary Fig.8. Glucocorticoid treatment of different breast cancer cell lines. The figures depict qPCR screening results for dexamethasone treatment of MCF-7 (ER+), MDA-MB-231 (TNBC), and HCC1954 (HER2+) breast cancer cell lines. *FKBP5* served as a positive control. * *P* < 0.05, ** *P* < 0.01, *** *P* < 0.001 from student’s t test.


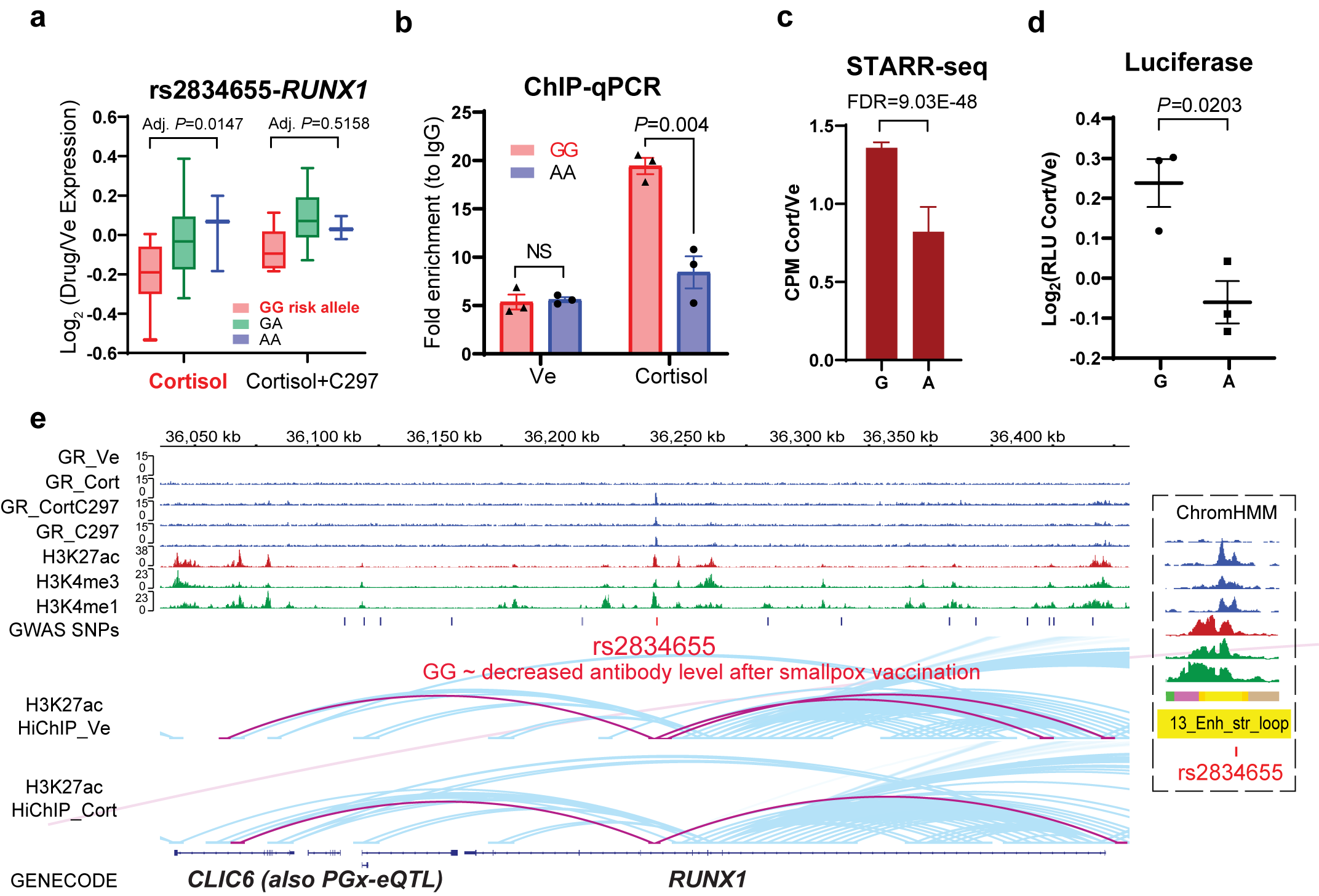


Supplementary Fig.9. Example of a disease-associated PGx-eQTL SNP-gene pair for which the intronic enhancer SNP loops to the promoter of the eQTL gene in which it resides. (a) PGx-eQTL SNP-gene pair for rs2834655-*RUNX1*. Adjusted *P*-values from Tukey’s post-hoc multiple comparisons and represent differences between wildtype and variant genotypes. Cortisol induced the eQTL, and C297 antagonized the cortisol effect, normalizing eQTL expression. (b) ChIP-qPCR of GR demonstrating PGx SNP-mediated disruption of GR binding after cortisol treatment. This effect correlated with (c) lower enhancer activity as measured by STARR-seq. (c) SNP-dependent and drug-dependent enhancer activity of the PGx locus as measured by STARR-seq. CPM stands for counts per million. (d) Luciferase reporter gene assay validated STARR-seq results. (e) IGV plots of the PGx SNP-eQTL gene locus. Tracks for GR-targeted ChIP-seq in different drug conditions are colored in blue, which show similar drug-dependent pattern as expression data: Cortisol induced GR binding at SNP locus, and C297 antagonized the cortisol effect, reducing GR binding. H3K4me1 is a histone mark associated with enhancers. H3K4me3 is a histone mark associated with promoters. H3K27ac is a histone mark associated with active promoters and enhancers. For the H3K27ac HiChIP tracks, loops directly interacting with the PGx SNP locus are highlighted in pink and others in blue.


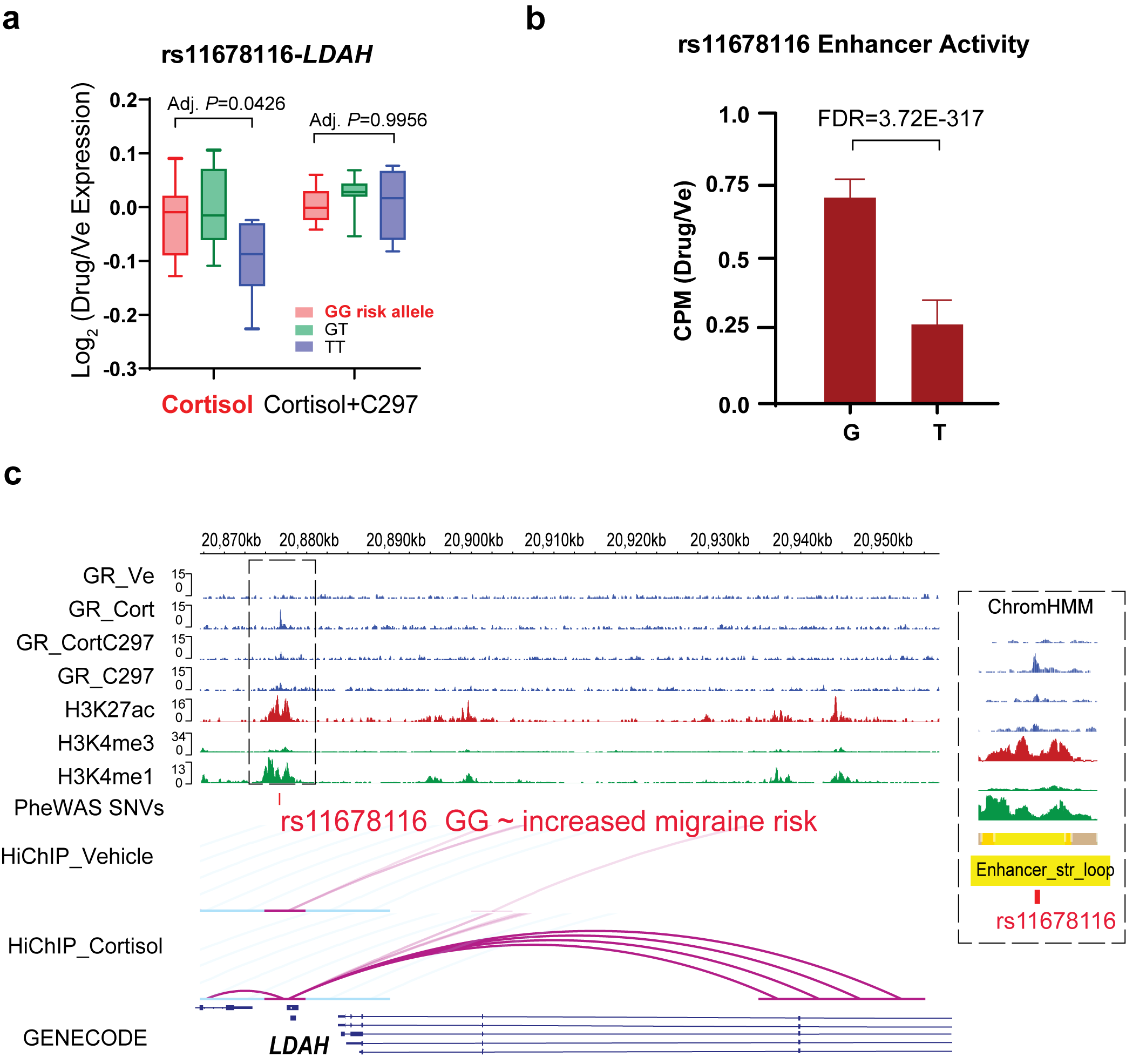


Supplementary Fig.10. Example of a disease-associated PGx-eQTL SNP-gene pair for which induction of loops by cortisol was observed, bringing the intergenic enhancer SNP and the eQTL gene together. (a) PGx-eQTL SNP-gene pair for rs11678116-*LDAH*. Adjusted *P*-values from Tukey’s post-hoc multiple comparisons represent differences between wildtype and variant genotypes. Cortisol induced the eQTL, and C297 antagonized the cortisol effect, normalizing eQTL expression. (b) SNP-dependent and drug-dependent enhancer activity of the PGx locus as measured by STARR-seq. CPM stands for counts per million. (c) IGV plots of the PGx SNP-eQTL gene locus.


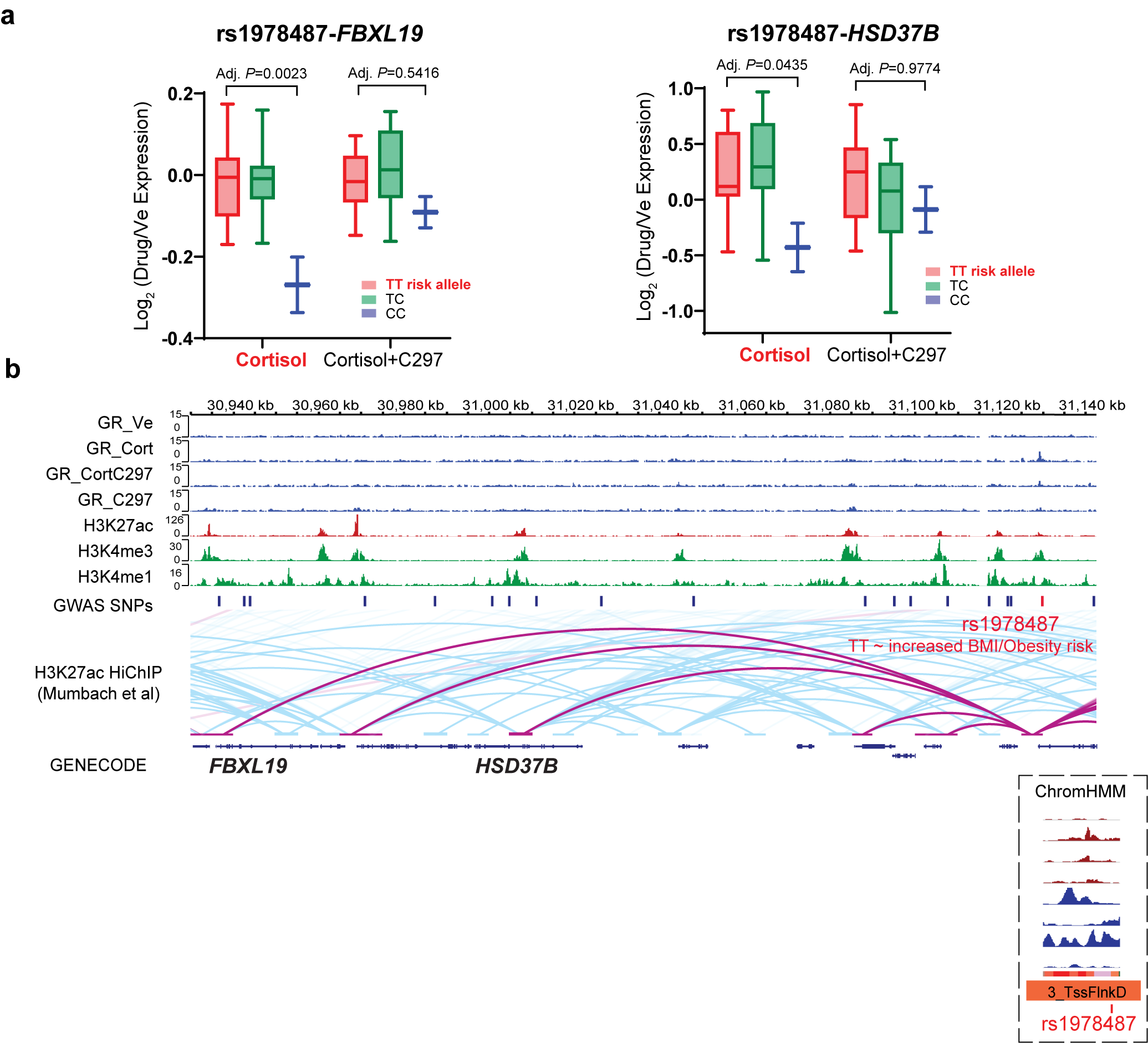


Supplementary Fig.11. Example of a disease-associated PGx-eQTL SNP-gene pair for which the promoter SNP of a gene loops to the promoter of the eQTL genes located far away. (a) PGx-eQTL SNP-gene pairs for rs1978487-*FBXL19*/*HSD37B*. Adjusted *P*-values from Tukey’s multiple comparisons represent differences between wildtype and variant genotypes. Cortisol induced the eQTL, and C297 antagonized the cortisol effect, normalizing eQTL expression for both genes in similar fashion across genotypes. (b) IGV plots of the PGx SNP-eQTL gene locus.

**Supplemetary Tables**

Supplementary Table 1. Information for the 30 LCLs used in this study

| **Cell ID** | **Catalog ID** | **Sex** | **Age (At sampling)** |
| --- | --- | --- | --- |
| CA01 | GM17201 | Male | 25 |
| CA07 | GM17207 | Male | 28 |
| CA12 | GM17212 | Male | 51 |
| CA14 | GM17214 | Male | 33 |
| CA15 | GM17215 | Female | 44 |
| CA18 | GM17218 | Female | 51 |
| CA21 | GM17221 | Female | 22 |
| CA36 | GM17236 | Female | 26 |
| CA38 | GM17238 | Female | 34 |
| CA40 | GM17240 | Male | 60 |
| CA41 | GM17241 | Male | 40 |
| CA45 | GM17245 | Female | 48 |
| CA49 | GM17249 | Male | 44 |
| CA50 | GM17250 | Female | 35 |
| CA56 | GM17256 | Female | 24 |
| CA58 | GM17258 | Female | 27 |
| CA60 | GM17260 | Female | 28 |
| CA61 | GM17261 | Female | 28 |
| CA63 | GM17263 | Female | 26 |
| CA64 | GM17264 | Male | 25 |
| CA65 | GM17265 | Female | 25 |
| CA66 | GM17266 | Female | 34 |
| CA68 | GM17268 | Male | 26 |
| CA69 | GM17269 | Male | 55 |
| CA79 | GM17279 | Female | 54 |
| CA84 | GM17284 | Female | 28 |
| CA86 | GM17286 | Female | 73 |
| CA89 | GM17289 | Female | 73 |
| CA93 | GM17293 | Female | 20 |
| CA94 | GM17294 | Female | 33 |

Supplementary Table 2. Full annotation of 25 chromatin states built from 15 epigenetic marks in LCLs

| **STATE NO.** | **STATE ANNOTATION** | **ABBRIEVIATION** | **COLOR** | **COLOR CODE** |
| --- | --- | --- | --- | --- |
| 1 | POLR2 initiation & stop, enhancer-looping | TssTes_loop | Red | 255,0,0 |
| 2 | Active TSS, 5' flank, enhancer-looping | TssFlnkU_loop | Orange Red | 255,69,0 |
| 3 | Active TSS, 3' flank, enhancer-looping | TssFlnkD_loop | Tomato | 255,99,71 |
| 4 | Flanks active TSS 5' and 3' | TssFlnkUD | Coral | 255,127,80 |
| 5 | Bivalent promoter | TssBiv | Salmon | 250,128,114 |
| 6 | Transcribed 5' proximal | Tx1 | Darkgreen | 0,100,0 |
| 7 | Transcribed less 5' proximal | Tx2 | Green | 0,128,0 |
| 8 | Transcribed 5' distal | Tx3 | ForestGreen | 34,139,34 |
| 9 | Weakly transcribed | Tx4 | LightGreen | 144,238,144 |
| 10 | Transcribed exons | TxExon | Lime | 0,255,0 |
| 11 | End of transcription | TxEnd | Limegreen | 50,205,50 |
| 12 | Enhancer | Enh | Gold | 255,215,0 |
| 13 | Strong enhancer, promoter-looping | Enh_str_loop | Yellow | 255,255,0 |
| 14 | Weak enhancer, promoter-looping | Enh_wk_loop | Khaki | 240,230,140 |
| 15 | Poised enhancer | EnhP | Tan | 210,180,140 |
| 16 | Poised enhancer, upstream of TSS | EnhP_TssU | Navajowhite | 255,222,173 |
| 17 | Genic enhancer | EnhG1 | DeepPink | 255,20,147 |
| 18 | Genic enhancer, weak, 3' of state #17 | EnhG2 | LightPink | 255,182,193 |
| 19 | Genic enhancer, flanks state #17 5' and 3' | EnhG3 | Violet | 238,180,238 |
| 20 | Open chromatin/TF-binding, near repressed | OpenTF | Blue | 0,0,255 |
| 21 | Insulator | Ins | Purple | 128,0,128 |
| 22 | Repeats | Rpts | DarkSlateGray | 47,79,79 |
| 23 | Quiescent/low signal 1 | Quies | Lightgray | 211,211,211 |
| 24 | Quiescent/low signal 2 | Quies | Gainsboro | 220,220,220 |
| 25 | Polycomb-repressed | ReprPC | Gray | 128,128,128 |

Supplementary Table 3. Distribution of PGx-eQTL SNPs in each chromatin state

**CORT**

| **Chromatin states** | **Counts of SNP-gene pair(s)** | |
| --- | --- | --- |
| 1_TssTes_loop | 1 | 1% |
| 10_TxExon | 1 | 1% |
| 12_Enh | 4 | 4% |
| 13_Enh_str_loop | 36 | 35% |
| 14_Enh_wk_loop | 6 | 6% |
| 15_EnhP | 11 | 11% |
| 16_EnhP_TssU | 1 | 1% |
| 17_EnhG1 | 6 | 6% |
| 18_EnhG2 | 6 | 6% |
| 19_EnhG3 | 12 | 12% |
| 20_OpenTF | 2 | 2% |
| 22_Rpts | 1 | 1% |
| 23_Quies | 7 | 7% |
| 25_ReprPC | 2 | 2% |
| 3_TssFlnkD_loop | 4 | 4% |
| 4_TssFlnkUD | 2 | 2% |
| Total | 102 | 100% |

**C297**

| **Chromatin states** | **Counts of SNP-gene pair(s)** | |
| --- | --- | --- |
| 13_Enh_str_loop | 19 | 59% |
| 14_Enh_wk_loop | 2 | 6% |
| 16_EnhP_TssU | 1 | 3% |
| 19_EnhG3 | 1 | 3% |
| 2_TssFlnkU_loop | 4 | 13% |
| 20_OpenTF | 1 | 3% |
| 3_TssFlnkD_loop | 4 | 13% |
| Grand Total | 32 | 100% |

Supplementary Table 4. Current knowledge of GR-dependent PGx-eQTL gene functions

| **PGx-eQTLs** | **Associated Disease(s)** | **Known Gene Function Based on Literature (not exhaustive list)** |
| --- | --- | --- |
| rs2834655 – *RUNX1* | Immune response to smallpox vaccine (1)  Red blood cell count (2)  Monocyte percentage of white cells (3) | Blood cell development & hematopoiesis (4-7) |
| rs2984920/  rs7535818 – *RGS1* | Systemic lupus erythematosus (8)  Intestinal malabsorption/Celilac disease (9)  Multiple sclerosis (10) | B-lymphocyte function (11), T helper cells function (12), chemokine signaling in atherosclerosis (13) |
| rs4735336 – *PLEKHF2* | Viral hepatitis C (9) | Unknown; interacts with SARS-CoV-2 protein ORF8 (14) |
| rs2297539 – *IKBKE & SRGAP2* | Fasciitis  Celilac disease (9,15) | *IKBKE* is an oncogene (16-18) and limits inflammation in blood (19); *SRGAP2* regulates neuronal development (20) |
| rs2051541– *HIST1H2AC* | Disorder of iron metabolism  Celilac disease (9,15) | Unknown |
| rs7356 – *EYA3* | Platelet count (21) | Cell proliferation (22); mediate tumor-associated immune suppression (23) |
| rs4843073 – *KLHL25* | Neutrophil count (3) | Inhibits lipid synthesis and tumor progression (24) |
| rs4984913 – *CCDC78/WDR90* | Plateletcrit (3) | Both are critical for centriole biogenesis (25,26) |
| rs2779180 – *ARRDC5* | Depression in response to interferon-based therapy in chronic hepatitis C (27) | Unknown |
| rs1050863 – *ZCCHC14 & JPH3* | Mood Instability (28) | *JPH3* mediates protein toxicity in Huntington’s disease (29); *ZCCHC14* regulates cell proliferation in lung cancer (30) |
| rs1697139 – *MAST4* | Breast cancer (31) | Unknown |
| rs9594738 – *DGKH* | Bone mineral density (32)  Medication use (drugs affecting bone structure and mineralization) (33) | Unknown; is a member of diaglycerol kinase family in which DGKζ is known to regulate osteoclast (34,35); has been associated with bipolar disorder (36) |
| rs1978487 – *FBXL19 & HSD3B7* | Obesity/hypertension/BMI (15) | *FBXL19* controls adipogenesis (37); *HSD3B7* controls bile acid synthesis from cholesterol (38) |
| rs7288411 – *RRP7B* | Bipolar disorder or major depressive disorder (39) | Unknown |
| rs11215427 – *CADM1* | Childhood body mass index (40) | Regulation of body weight and energy homeostasis through CNS (41); Insulin secretion (42) |
| rs2399594–*NLRC5* | Lichen Planus (inflammation of the skin) | Key regulator of MHC class I-dependent immune responses (43) |

Supplementary Data (Excel Sheets)

Data S1. (excel file) Differentially expressed genes after glucocorticoids treatment in 30 LCLs

Data S2. (excel file) Integrated information for all identified glucocorticoid-dependent PGx-eQTL SNP-gene pairs

Data S3. (excel file) ENCODE epigenetic datasets generated in LCL used for ChromHMM analysis

Data S4. (excel file) Primer sequences for STARR-seq locus library

Supplementary Methods

**Glucocorticoid-targeted chromatin immunoprecipitation (ChIP)-sequencing library preparation**

Cell pellets were washed with ice-cold Tris-buffered saline (TBS). The cell pellet was then resuspended in cell lysis buffer (10mM Tris HCl, pH7.5, 10 mM NaCl, 0.5% IGEPAL), and was incubated on ice for 10 min. The lysates were then washed with MNase digestion buffer (20 mM Tris-HCl, pH 7.5, 15 mM NaCl, 60 mM KCl, 1 mM CaCl_2_), and incubated with MNase (2000 gel units per 4×10^6^ cells, New England Biolabs) for 20 min at 37 °C. The reaction was stopped by the addition of the same volume of sonication buffer (100 mM Tris-HCl, pH8.1, 20 mM EDTA, 200 mM NaCl, 2% Triton X-100, 0.2% Sodium deoxycholate). The lysate was then sonicated for 10 cycles (30s on, 30s off) with a Diagenode Bioruptor, and centrifuged at 15,000 rpm for 10 min at 4°C. A final concentration of 0.05% SDS was added to the supernatant to reduce the background. A cocktail of antibodies against the glucocorticoid receptor was added to chromatin input for immunoprecipitation: 2 mg of ab3570 (Abcam, lot GR3222141-5, discontinued) and 0.49 mg of 12041S (Cell Signaling Technology, lot 3) per 20 million cells. The reaction was incubated at 4 °C on a rocker overnight. On the next day, 30 μl of pre-washed protein G-magnetic beads was added to the reaction and incubated for 3 h. The beads were extensively washed with ChIP buffer (100 mM Tris-HCl, pH 8.1, 20 mM EDTA, 200 mM NaCl, 2% Triton X-100, 0.2% Sodium deoxycholate), twice with high salt buffer (ChIP buffer + 0.5 M NaCl), twice with LiCl_2_ buffer (0.25M LiCl_2_ in 10mM Tris-HCl, pH 8.1), and twice with TE buffer (10mM Tris-HCl, pH 8.1 and 1 mM EDTA). All washes were carried out for 5 mins at room temperature on a rotating wheel. Bound chromatin was eluted twice with 50 μL ChIP elution buffer (10 mM Tris-HCl, pH 8.0, 10 mM EDTA, 150 mM NaCl, 5 mM DTT, 1% SDS) and reverse-crosslinked at 65 °C overnight, including input. On the next day, 2 ml of DNase-free RNase A (10 mg/ml, Thermo Scientific, Cat. #: EN0531) was added per 100mL eluent, and was incubated at 37 °C for 1 hour. 10mL proteinase K (20 mg/ml, Ambion, Cat. #: AM2546) was added per 100mL eluent and was incubated at 37 °C for 3 hours. DNA was then purified with the MinElute PCR Purification Kit (Qiagen, Cat. #28006) per manufacturer’s instructions, and quantified using the Qubit dsDNA High Sensitivity assay (Invitrogen, Cat. # Q32851). The size of purified DNA was analyzed by the Fragment Analyzer (Advanced Analytical Technologies) using the High Sensitivity NGS Fragment Analysis Kit (Cat. # DNF-486). ChIP-seq libraries were prepared for sequencing with the ThruPLEX® DNA-seq Kit V2 (Rubicon Genomics) according to the manufacturer’s instructions.

**Massively parallel reporter assay (STARR-seq) experiments**

***A. Input library construction***

Human STARR-seq-ORI vector was obtained from Addgene (plasmid #99296). 8ug of STARR-seq plasmid was digested with 20uL AgeI-HF® (R3552, NEB), 20uL SalI-HF® (R3138, NEB), and 8uL Thermosensitive Alkaline Phosphatase (TSAP) in Multicore10X Buffer (Promega). Digestion was performed at 37 °C for 16 hours, followed by inactivation of TSAP at 74°C for 15 minutes. The digested products were then purified with QIAquick Gel Extraction Kit and Qiagen MinElute PCR purification kits. For inserts, we amplified all identified PGx-eQTL peaks extended by +/- 500bp using genomic DNA extracted from the 30 LCLs (**Table S1**). Primers were designed with the Nebuilder Assembly Tool v2.2.7. If the beginning or end of a region was difficult to anneal, we extended the region to include 50-100bp at the 3’ or 5’ end (for primers sequences and specific PCR conditions, refer to **Supplementary** **Data 4**). After amplification, every 10 PCR products were pooled together, gel purified, and cloned into the STARR-seq plasmid with NEBuilder® HiFi DNA Assembly Cloning Kit (E5520S, New England Biolabs) per manufacturer’s instruction. The ratio of vector (~3000bp) to inserts (~1200bp) was 0.05 pmol to 0.15 pmol. We included in the library a GR-induced peak within a strong enhancer region near the promoter of *FKBP5*, extended by +/-500bp, as a positive control. Assembled DNA products were then chemically transformed into NEB^®^ 5-alpha competent *E. coli* and spread on LB-ampicillin agar plates. The plates were scraped and grown in 2 liters of LB-ampicillin. Plasmids were extracted with the HiSpeed Plasmid Maxi Kit (Qiagen).

To check the quality of the inserted DNA loci (input library), 10ng plasmid DNA was amplified with KAPA HiFi HotStart ReadyMix PCR Kit (KK2601, KAPABiosystem) using universal primers TTCTCTCCACAGGTGTCCA (forward) and GCAATAGCATCACAAATTTCACA (reverse) with the following conditions: 95°C for 3 minutes, followed by 9 cycles of 98°C for 20 seconds, 63°C for 15 seconds, and 72°C for 70 seconds. The PCR products were purified with QIAquick Gel Extraction Kit and Qiagen MinElute PCR purification kits and were then sequenced on a Hiseq4000 (Illumina) with 150-bp paired-end sequencing.

***B. Transfection of input libraries into two cell lines***

Due to their sensitivity to FBS, LCLs were not grown in charcoal-stripped media for this set of electroporation experiment as cell viability is a critical factor for successful STARR-seq screening. Therefore, to better evaluate GR activity on PGx loci cloned in STARR-seq plasmids, we also included A549, a lung cancer cell line in which GR has been extensively studied (44), in our STARR-seq screening. LCLs and A549 were maintained at viability of > 90% before transfection. For LCLs, 8 million cells were transfected with 20ug of plasmid DNA in one 100-uL cuvette using the Amaxa® Cell Line Nucleofector® Kit V (Lonza), program X-001. 10 independent pulses per drug condition were conducted, which resulted in a total of 600ug/240 million cells. For A549, 5 million cells were transfected with 10ug of plasmid DNA in one 100-uL cuvette using the Amaxa® Cell Line Nucleofector® Kit T (Lonza), program X-001. 15 independent pulses per drug condition were conducted, which resulted in a total of 450ug/225 million cells. After electroporation, cells were treated with drugs (hydrocortisone, C297 or vehicle) in 5% CS media for 9 hours. Total RNA was then harvested with the RNA Maxiprep kit per manufacturer’s instructions (Qiagen).

***C. RNA library preparation and sequencing***

Messenger RNA isolation was conducted with the Dynabeads Oligo (dT)_25_ (Invitrogen), followed by TURBO™ DNase digestion. The reaction was then cleaned up with RNA CleanXP beads (Beckman). mRNA concentrations were measured with Nanodrop 8000. We conducted first-strand cDNA synthesis with the SuperScript III Reverse Transcript kit (Invitrogen) using a reporter transcript-specific primer CTCATCAATGTATCTTATCATGTCTG and no more than 500ng mRNA per reaction. cDNA pool was then treated with RNAseA and purified with AMPure XP beads (Beckman). We conducted junction PCR with the KAPA HiFi HotStart ReadyMix PCR Kit (KK2601, KAPABiosystem) using primers TCGTGAGGCACTGGGCAG*G*T*G*T*C (forward) and CTTATCATGTCTGCTCGA*A*G*C (reverse) with the following conditions: 98°C for 45 seconds, followed by 16 cycles of 98°C for 15 seconds, 65°C for 30 seconds, and 72°C for 70 seconds. A total of 20 and 30 PCR reactions were conducted for LCLs and A549 cells, respectively. The PCR products were purified with AMPure XP beads (Beckman) before being sheared, adapter-ligated with NEBNext® Ultra™ DNA Library Prep Kit, and sequenced on an Illumina Hiseq 4000 with pair-end mode 2x150bp.

**Functional validation of selected disease-associated PGx-eQTL experiments**

***Glucocorticoid-targeted ChIP-qPCR***.

To test GR activity for the rs1697139-*MAST4* PGx-eQTL locus in a breast cancer cell line, MDA-MB-231 cells were steroid-starved for 48 hours and subjected to vehicle and 100nM dexamethasone treatment for 1.5 hours (dexamethasone instead of cortisol was used to avoid cortisol effect on the mineralocorticoid receptor since, unlike LCLs, MDA-MB-231 expressed the mineralocorticoid receptor at reasonable levels). Similar ChIP experiment conditions were conducted as described above. Two sets of primers targeting the rs1697139 GR-binding locus were used: Forward CACCTCAAAGGGTCCTCGGT, reverse TGTCACCACTTTCAGTTCCCTA (78 bp amplicon), and forward AGTGGTGACAACAGAGGATGTG, reverse TGTTCCTCGTCAGAAGCAGTT (79 bp amplicon).

To test SNP-dependent GR activity for rs12834655-*RUNX1* PGx-eQTL locus, GM17215 (rs12834655 genotype AA) and GM17293 (rs12834655 genotype GG) cells were steroid starved for 48 hours and subjected to vehicle and 100nM cortisol treatment for 1.5 hours. The chromatin immunoprecipitation steps and conditions were conducted as described above in the glucocorticoid-targeted ChIP-sequencing section. The Rabbit IgG control antibodies for ChIP-qPCR experiments were ab172730 (abcam, GR3298823-2, 2ug per 80ug chromatin), and Normal IgG Rabbit 2729 (Cell Signaling, lot 3, 0.49ug per 80ug chromatin). Quantitative PCR primers were designed with the NCBI Primers Blast Tools to ensure specificity of primers against the whole genome. The product size was 78bp, located inside the GR peak. The forward primer sequence was TTAGTCTTGACGTTGGCGGC, and reverse was GGTTCCAGCCGTGGCTTTAT. A primer targeting a GR-inducible peak upstream of *FKBP5* was used as a positive control similar to that in STARR-seq studies. The forward primer for this sequence was AGCGGGGGTTCTAGAGAGTG and reverse primer was CCAGGTTCTCAGGATCTCGT. The product size was 77bp. Each qPCR reaction was conducted with the *Power* SYBR™ Green PCR Master Mix (Thermo Fisher, Cat# 4367659) in triplicate. The Ct numbers of ChIP DNA were then normalized to Ct numbers of input DNA, and then normalized to Ct numbers of IgG. Data graphs were plotted using Prism (GraphPad Software). Statistical comparisons between genotypes were made using two-tailed student’s t-test.

***Single-cell selection for CRISPR/Cas9 edited cells***

A total of 1000 edited MDA-MB-231 bulk cells were cultured in a 100mm cell culture plates for 1.5 weeks. Two 96-well cell culture plates were then prepared before single-colony selection – one for genotyping and one for maintaining the cells. In the genotyping plate, 20 uL Trypsin (0.25% EDTA) was added in each well. In the maintaining plate, 100uL of cell culture medium was added in each well. From the 100mm plate, each colony was picked up by pipette and digested for 5 minutes at 37°C. 100uL of cell culture medium was added to neutralize trypsin and cells were pipetted up and down for several times to make single cell suspension. 30uL of this solution was transferred into the maintain plate to keep growing. The genotyping plate were grown for 48h before cells were lysed for genotyping. Medium in wells were removed and 20uL of cell lysis buffer was added. The recipe for cell lysis was previously described (45). The wells were covered by shipping tape and incubated at 55°C for 1 hour. The lysate was then transferred into a 96-well PCR plate and incubated for 10min at 95°C. 1uL of cell lysis from the PCR plate was used as the template for the PCR reaction of genotyping. The PCR condition and primers were the same as described in the main text. Colonies were selected from the 96-well maintaining plate, expanded and re-genotyped before treatment experiment.

**References**

1. Ovsyannikova, I.G., Kennedy, R.B., O'Byrne, M., Jacobson, R.M., Pankratz, V.S. and Poland, G.A. (2012) Genome-wide association study of antibody response to smallpox vaccine. *Vaccine*, **30**, 4182-4189.

2. Kichaev, G., Bhatia, G., Loh, P.R., Gazal, S., Burch, K., Freund, M.K., Schoech, A., Pasaniuc, B. and Price, A.L. (2019) Leveraging Polygenic Functional Enrichment to Improve GWAS Power. *Am J Hum Genet*, **104**, 65-75.

3. Vuckovic, D., Bao, E.L., Akbari, P., Lareau, C.A., Mousas, A., Jiang, T., Chen, M.H., Raffield, L.M., Tardaguila, M., Huffman, J.E. *et al.* (2020) The Polygenic and Monogenic Basis of Blood Traits and Diseases. *Cell*, **182**, 1214-1231 e1211.

4. Chi, Y., Huang, Z., Chen, Q., Xiong, X., Chen, K., Xu, J., Zhang, Y. and Zhang, W. (2018) Loss of runx1 function results in B cell immunodeficiency but not T cell in adult zebrafish. *Open Biol*, **8**.

5. Sood, R., Kamikubo, Y. and Liu, P. (2017) Role of RUNX1 in hematological malignancies. *Blood*, **129**, 2070-2082.

6. Bonifer, C., Levantini, E., Kouskoff, V. and Lacaud, G. (2017) Runx1 Structure and Function in Blood Cell Development. *Adv Exp Med Biol*, **962**, 65-81.

7. Mevel, R., Draper, J.E., Lie, A.L.M., Kouskoff, V. and Lacaud, G. (2019) RUNX transcription factors: orchestrators of development. *Development*, **146**.

8. Langefeld, C.D., Ainsworth, H.C., Cunninghame Graham, D.S., Kelly, J.A., Comeau, M.E., Marion, M.C., Howard, T.D., Ramos, P.S., Croker, J.A., Morris, D.L. *et al.* (2017) Transancestral mapping and genetic load in systemic lupus erythematosus. *Nat Commun*, **8**, 16021.

9. Gagliano Taliun, S.A., VandeHaar, P., Boughton, A.P., Welch, R.P., Taliun, D., Schmidt, E.M., Zhou, W., Nielsen, J.B., Willer, C.J., Lee, S. *et al.* (2020) Exploring and visualizing large-scale genetic associations by using PheWeb. *Nat Genet*, **52**, 550-552.

10. Andlauer, T.F., Buck, D., Antony, G., Bayas, A., Bechmann, L., Berthele, A., Chan, A., Gasperi, C., Gold, R., Graetz, C. *et al.* (2016) Novel multiple sclerosis susceptibility loci implicated in epigenetic regulation. *Sci Adv*, **2**, e1501678.

11. Moratz, C., Kang, V.H., Druey, K.M., Shi, C.S., Scheschonka, A., Murphy, P.M., Kozasa, T. and Kehrl, J.H. (2000) Regulator of G protein signaling 1 (RGS1) markedly impairs Gi alpha signaling responses of B lymphocytes. *J Immunol*, **164**, 1829-1838.

12. Caballero-Franco, C. and Kissler, S. (2016) The autoimmunity-associated gene RGS1 affects the frequency of T follicular helper cells. *Genes Immun*, **17**, 228-238.

13. Patel, J., McNeill, E., Douglas, G., Hale, A.B., de Bono, J., Lee, R., Iqbal, A.J., Regan-Komito, D., Stylianou, E., Greaves, D.R. *et al.* (2015) RGS1 regulates myeloid cell accumulation in atherosclerosis and aortic aneurysm rupture through altered chemokine signalling. *Nat Commun*, **6**, 6614.

14. Gordon, D.E., Jang, G.M., Bouhaddou, M., Xu, J., Obernier, K., White, K.M., O'Meara, M.J., Rezelj, V.V., Guo, J.Z., Swaney, D.L. *et al.* (2020) A SARS-CoV-2 protein interaction map reveals targets for drug repurposing. *Nature*, **583**, 459-468.

15. McInnes, G., Tanigawa, Y., DeBoever, C., Lavertu, A., Olivieri, J.E., Aguirre, M. and Rivas, M.A. (2019) Global Biobank Engine: enabling genotype-phenotype browsing for biobank summary statistics. *Bioinformatics*, **35**, 2495-2497.

16. Yin, M., Wang, X. and Lu, J. (2020) Advances in IKBKE as a potential target for cancer therapy. *Cancer Med*, **9**, 247-258.

17. Rajurkar, M., Dang, K., Fernandez-Barrena, M.G., Liu, X., Fernandez-Zapico, M.E., Lewis, B.C. and Mao, J. (2017) IKBKE Is Required during KRAS-Induced Pancreatic Tumorigenesis. *Cancer Res*, **77**, 320-329.

18. Boehm, J.S., Zhao, J.J., Yao, J., Kim, S.Y., Firestein, R., Dunn, I.F., Sjostrom, S.K., Garraway, L.A., Weremowicz, S., Richardson, A.L. *et al.* (2007) Integrative genomic approaches identify IKBKE as a breast cancer oncogene. *Cell*, **129**, 1065-1079.

19. Patel, M.N., Bernard, W.G., Milev, N.B., Cawthorn, W.P., Figg, N., Hart, D., Prieur, X., Virtue, S., Hegyi, K., Bonnafous, S. *et al.* (2015) Hematopoietic IKBKE limits the chronicity of inflammasome priming and metaflammation. *Proc Natl Acad Sci U S A*, **112**, 506-511.

20. Fossati, M., Pizzarelli, R., Schmidt, E.R., Kupferman, J.V., Stroebel, D., Polleux, F. and Charrier, C. (2016) SRGAP2 and Its Human-Specific Paralog Co-Regulate the Development of Excitatory and Inhibitory Synapses. *Neuron*, **91**, 356-369.

21. Chen, M.H., Raffield, L.M., Mousas, A., Sakaue, S., Huffman, J.E., Moscati, A., Trivedi, B., Jiang, T., Akbari, P., Vuckovic, D. *et al.* (2020) Trans-ethnic and Ancestry-Specific Blood-Cell Genetics in 746,667 Individuals from 5 Global Populations. *Cell*, **182**, 1198-1213 e1114.

22. Ionescu, A.E., Mentel, M., Munteanu, C.V.A., Sima, L.E., Martin, E.C., Necula-Petrareanu, G. and Szedlacsek, S.E. (2019) Analysis of EYA3 Phosphorylation by Src Kinase Identifies Residues Involved in Cell Proliferation. *Int J Mol Sci*, **20**.

23. Vartuli, R.L., Zhou, H., Zhang, L., Powers, R.K., Klarquist, J., Rudra, P., Vincent, M.Y., Ghosh, D., Costello, J.C., Kedl, R.M. *et al.* (2018) Eya3 promotes breast tumor-associated immune suppression via threonine phosphatase-mediated PD-L1 upregulation. *J Clin Invest*, **128**, 2535-2550.

24. Zhang, C., Liu, J., Huang, G., Zhao, Y., Yue, X., Wu, H., Li, J., Zhu, J., Shen, Z., Haffty, B.G. *et al.* (2016) Cullin3-KLHL25 ubiquitin ligase targets ACLY for degradation to inhibit lipid synthesis and tumor progression. *Genes Dev*, **30**, 1956-1970.

25. Klos Dehring, D.A., Vladar, E.K., Werner, M.E., Mitchell, J.W., Hwang, P. and Mitchell, B.J. (2013) Deuterosome-mediated centriole biogenesis. *Dev Cell*, **27**, 103-112.

26. Steib, E., Laporte, M.H., Gambarotto, D., Olieric, N., Zheng, C., Borgers, S., Olieric, V., Le Guennec, M., Koll, F., Tassin, A.M. *et al.* (2020) WDR90 is a centriolar microtubule wall protein important for centriole architecture integrity. *Elife*, **9**.

27. Matsunami, K., Nishida, N., Kaneko, N., Ikeo, K., Toyo-Oka, L., Takeuchi, H., Matsuura, K., Tamori, A., Nomura, H., Yoshiji, H. *et al.* (2016) Genome-Wide Association Study Identifies ZNF354C Variants Associated with Depression from Interferon-Based Therapy for Chronic Hepatitis C. *PLoS One*, **11**, e0164418.

28. Ward, J., Tunbridge, E.M., Sandor, C., Lyall, L.M., Ferguson, A., Strawbridge, R.J., Lyall, D.M., Cullen, B., Graham, N., Johnston, K.J.A. *et al.* (2019) The genomic basis of mood instability: identification of 46 loci in 363,705 UK Biobank participants, genetic correlation with psychiatric disorders, and association with gene expression and function. *Mol Psychiatry*.

29. Wilburn, B., Rudnicki, D.D., Zhao, J., Weitz, T.M., Cheng, Y., Gu, X., Greiner, E., Park, C.S., Wang, N., Sopher, B.L. *et al.* (2011) An antisense CAG repeat transcript at JPH3 locus mediates expanded polyglutamine protein toxicity in Huntington's disease-like 2 mice. *Neuron*, **70**, 427-440.

30. Shi, X., Han, X., Cao, Y., Li, C. and Cao, Y. (2021) ZCCHC14 regulates proliferation and invasion of non-small cell lung cancer through the MAPK-P38 signalling pathway. *J Cell Mol Med*, **25**, 1406-1414.

31. Michailidou, K., Lindstrom, S., Dennis, J., Beesley, J., Hui, S., Kar, S., Lemacon, A., Soucy, P., Glubb, D., Rostamianfar, A. *et al.* (2017) Association analysis identifies 65 new breast cancer risk loci. *Nature*, **551**, 92-94.

32. Styrkarsdottir, U., Halldorsson, B.V., Gretarsdottir, S., Gudbjartsson, D.F., Walters, G.B., Ingvarsson, T., Jonsdottir, T., Saemundsdottir, J., Center, J.R., Nguyen, T.V. *et al.* (2008) Multiple genetic loci for bone mineral density and fractures. *N Engl J Med*, **358**, 2355-2365.

33. Wu, Y., Byrne, E.M., Zheng, Z., Kemper, K.E., Yengo, L., Mallett, A.J., Yang, J., Visscher, P.M. and Wray, N.R. (2019) Genome-wide association study of medication-use and associated disease in the UK Biobank. *Nature Communications*, **10**.

34. Iwazaki, K., Tanaka, T., Hozumi, Y., Okada, M., Tsuchiya, R., Iseki, K., Topham, M.K., Kawamae, K., Takagi, M. and Goto, K. (2017) DGKzeta Downregulation Enhances Osteoclast Differentiation and Bone Resorption Activity Under Inflammatory Conditions. *J Cell Physiol*, **232**, 617-624.

35. Zamani, A., Decker, C., Cremasco, V., Hughes, L., Novack, D.V. and Faccio, R. (2015) Diacylglycerol Kinase zeta (DGKzeta) Is a Critical Regulator of Bone Homeostasis Via Modulation of c-Fos Levels in Osteoclasts. *J Bone Miner Res*, **30**, 1852-1863.

36. Baum, A.E., Akula, N., Cabanero, M., Cardona, I., Corona, W., Klemens, B., Schulze, T.G., Cichon, S., Rietschel, M., Nothen, M.M. *et al.* (2008) A genome-wide association study implicates diacylglycerol kinase eta (DGKH) and several other genes in the etiology of bipolar disorder. *Mol Psychiatry*, **13**, 197-207.

37. Acharya, A., Berry, D.C., Zhang, H., Jiang, Y., Jones, B.T., Hammer, R.E., Graff, J.M. and Mendell, J.T. (2019) miR-26 suppresses adipocyte progenitor differentiation and fat production by targeting Fbxl19. *Genes Dev*, **33**, 1367-1380.

38. Shea, H.C., Head, D.D., Setchell, K.D. and Russell, D.W. (2007) Analysis of HSD3B7 knockout mice reveals that a 3alpha-hydroxyl stereochemistry is required for bile acid function. *Proc Natl Acad Sci U S A*, **104**, 11526-11533.

39. Coleman, J.R.I., Gaspar, H.A., Bryois, J., Bipolar Disorder Working Group of the Psychiatric Genomics, C., Major Depressive Disorder Working Group of the Psychiatric Genomics, C. and Breen, G. (2020) The Genetics of the Mood Disorder Spectrum: Genome-wide Association Analyses of More Than 185,000 Cases and 439,000 Controls. *Biol Psychiatry*, **88**, 169-184.

40. Vogelezang, S., Bradfield, J.P., Ahluwalia, T.S., Curtin, J.A., Lakka, T.A., Grarup, N., Scholz, M., van der Most, P.J., Monnereau, C., Stergiakouli, E. *et al.* (2020) Novel loci for childhood body mass index and shared heritability with adult cardiometabolic traits. *PLoS Genet*, **16**, e1008718.

41. Rathjen, T., Yan, X., Kononenko, N.L., Ku, M.C., Song, K., Ferrarese, L., Tarallo, V., Puchkov, D., Kochlamazashvili, G., Brachs, S. *et al.* (2017) Regulation of body weight and energy homeostasis by neuronal cell adhesion molecule 1. *Nat Neurosci*, **20**, 1096-1103.

42. Zhang, C., Caldwell, T.A., Mirbolooki, M.R., Duong, D., Park, E.J., Chi, N.W. and Chessler, S.D. (2016) Extracellular CADM1 interactions influence insulin secretion by rat and human islet beta-cells and promote clustering of syntaxin-1. *Am J Physiol Endocrinol Metab*, **310**, E874-885.

43. Kobayashi, K.S. and van den Elsen, P.J. (2012) NLRC5: a key regulator of MHC class I-dependent immune responses. *Nat Rev Immunol*, **12**, 813-820.

44. Reddy, T.E., Pauli, F., Sprouse, R.O., Neff, N.F., Newberry, K.M., Garabedian, M.J. and Myers, R.M. (2009) Genomic determination of the glucocorticoid response reveals unexpected mechanisms of gene regulation. *Genome Res*, **19**, 2163-2171.

45. Nussbaum, L., Telenius, J.M., Hill, S., Hirschfeld, P.P., Suciu, M.C., Consortium, W., Downes, D.J. and Hughes, J.R. (2018) High-Throughput Genotyping of CRISPR/Cas Edited Cells in 96-Well Plates. *Methods Protoc*, **1**.
